# High quality mapping of chromatin at or near the nuclear lamina from small numbers of cells reveals cell cycle and developmental changes of chromatin at the nuclear periphery

**DOI:** 10.1093/nar/gkac762

**Published:** 2022-09-21

**Authors:** Joseph R Tran, Xiaobin Zheng, Stephen A Adam, Robert D Goldman, Yixian Zheng

**Affiliations:** Department of Embryology, Carnegie Institution for Science, 3520 San Martin Drive, Baltimore, MD 21218, USA; Department of Embryology, Carnegie Institution for Science, 3520 San Martin Drive, Baltimore, MD 21218, USA; Department of Cell and Developmental Biology, Northwestern University, Feinberg School of Medicine, Ward Building 11-145, 303 E. Chicago Ave. Chicago, IL 60611, USA; Department of Cell and Developmental Biology, Northwestern University, Feinberg School of Medicine, Ward Building 11-145, 303 E. Chicago Ave. Chicago, IL 60611, USA; Department of Embryology, Carnegie Institution for Science, 3520 San Martin Drive, Baltimore, MD 21218, USA

## Abstract

The chromatin associated with the nuclear lamina (NL) is referred to as lamina-associated domains (LADs). Here, we present an adaptation of the tyramide-signal amplification sequencing (TSA-seq) protocol, which we call chromatin pull down-based TSA-seq (cTSA-seq), that can be used to map chromatin regions at or near the NL from as little as 50 000 cells. The cTSA-seq mapped regions are composed of previously defined LADs and smaller chromatin regions that fall within the Hi-C defined B-compartment containing nuclear peripheral heterochromatin. We used cTSA-seq to map chromatin at or near the assembling NL in cultured cells progressing through early G1. cTSA-seq revealed that the distal ends of chromosomes are near or at the reassembling NL during early G1, a feature similar to those found in senescent cells. We expand the use of cTSA-seq to the mapping of chromatin at or near the NL from fixed-frozen mouse cerebellar tissue sections. This mapping reveals a general conservation of NL-associated chromatin and identifies global and local changes during cerebellar development. The cTSA-seq method reported here is useful for analyzing chromatin at or near the NL from small numbers of cells derived from both *in vitro* and *in vivo* sources.

## INTRODUCTION

A portion of the metazoan genome is organized into compact, transcriptionally repressed DNA known as heterochromatin. In most cell types, a major fraction of heterochromatin is localized to the nuclear periphery and may directly interact with a protein meshwork known as the nuclear lamina (NL) that contains the filamentous A- and B-type lamins (1). The heterochromatin that directly interacts with the NL is known as lamina-associated domains (LADs) (2). A comparison of LADs and the A/B compartments defined from Hi-C experiments showed that LADs are in the B compartment, which is defined as ‘closed’ chromatin (2–4). The A and B compartments are, however, complex and each compartment can be further divided into sub-compartments. For example, the B compartment can be divided into sub-compartments B1 through B4 that are differentially enriched for epigenetic marks (e.g. H3K27me3 and H3K9me3) (4). Of these B sub-compartments, the B3 compartment shows the strongest correlation with LADs followed by the B2 compartment, which is correlated with both LADs and pericentromeric heterochromatin (4). The B1 compartment appears to be facultative heterochromatin, as it is enriched for H3K27me3 and depleted of H3K36me3, while the B4 compartment represents a small fraction of the genome (0.4% of the total) that is present only on chromosome 19 (4). Additional studies are needed to reveal if any chromatin in B compartments not mapped by LADs are near the NL. Mapping methods that identify chromatin at or near the NL would facilitate these investigations.

LADs are known to vary according to cell type (5,6) and recent work reveals that LADs, while largely stable, show dynamics during the cell cycle (7,8). The greatest dynamism has been reported during early G1 with an initial enrichment of NL-chromatin contacts occurring at the distal ends of chromosomes (7). These observations are consistent with previous studies of nuclear envelope reformation on decondensing post-mitotic chromosomes (9) and the reported increased spatial dynamics of genomic features, particularly those of telomeres, in early interphase (10–17). Collectively, these observations suggest a model where the distal ends of chromosomes transiently localize near the assembling NL in early G1 reminiscent of a Rabl-like configuration followed by re-organization and formation of a stable and typical LADs configuration.

LADs have been mapped by a variety of methods including chromatin-immunoprecipitation (ChIP)-seq, DNA adenine methyltransferase (Dam) identification (DamID), tyramide signal amplification (TSA)-sequencing (TSA-seq), ascorbate peroxidase 2 (APEX2) identification (APEX2-ID), and genome organization using CUT and RUN technology (GO-CaRT) (2,8,18–22). LADs were first mapped by DamID, which uses a fusion protein of bacterial Dam and lamin-B1 expressed at low levels in tissue culture cells (2). DNA located at the NL is methylated by contacting the Dam enzyme and can be identified by sequencing or microarray technologies. A modified form of this method can also be used to visualize LADs (16). The TSA-seq method employs a different strategy and uses antibody staining (e.g. anti-lamin-A/C) to specifically label the NL in fixed cells. A corresponding horseradish peroxidase (HRP)-conjugated secondary antibody then catalyzes the covalent attachment of biotin onto nearby proteins and DNA (18). The TSA-seq method uses a genomic DNA input where the fraction of biotinylated DNA is isolated by Streptavidin-bead pull down, purified and then subject to sequencing. Considering the diffusive nature of the TSA reaction, this method is useful both as a mapping tool and as a ‘cytological ruler’ and variations of this method have also found utility in proteomics (18,23). The GO-CaRT method, a new adaptation of the CUT and RUN technology, uses antibody targeting of protein A-MNase to map LADs *in vivo* and *in vitro* (22).

We recently adapted the use of the engineered APEX2 enzyme as a genome mapping tool, which we termed APEX2-identification (APEX2-ID), to identify stable and variable chromatin regions at or near the NL during the cell cycle (8,24,25). The APEX2-ID method uses the highly active APEX2 enzyme to catalyze the deposition of biotin onto proteins near the NL-localized fusion protein, which can then be isolated after chromatin fragmentation by pull down using Streptavidin beads. APEX2-ID can also be used to map NL-proximal proteins and associated RNAs from live cells and the activity of this enzyme persists under different conditions including cellular fixation. The van Steensel group recently developed an approach called protein-A DamID (pA-DamID) to map LADs from different cell cycle stages (7). The pA-DamID approach leverages antibody targeting, in a manner similar to TSA-seq, of DamID and revealed LADs dynamics during the cell cycle. The approach also revealed the enrichment of NL-chromatin interactions toward the distal ends of chromosomes in the early stage of G1. The pA-DamID, GO-CaRT and TSA-seq approaches highlight the utility of antibody-based methods.

Pros and cons exist for these aforementioned methods. For instance, the APEX2-ID method is a multi-purpose tool useful for mapping genomic regions, proteins and RNAs and the APEX2 reaction can be completed in a matter of seconds, thus allowing high-temporal resolution. The APEX2-ID approach is, however, limited by transfection issues and unwelcomed overexpression effects. DamID is a classic straightforward method that directly analyzes DNA, and is the only LADs mapping method for which single cell mapping has been reported (16,26). However, DamID requires transfection/transduction and several hours to sufficiently methylate DNA as the fusion protein is expressed at low levels (26). The recently reported GO-CaRT method can be used for *in vivo* and *in vitro* isolated live cells but can be complicated by structural damage during nuclei preparation from unfixed cells (22). The TSA-seq approach, as described by the Belmont group, is attractive as it allows the user to temporally resolve events based on the timing of cellular fixation and employs common immunostaining approaches familiar to most labs (18). The method, however, uses genomic DNA as an input source, and the fraction of DNA that is biotinylated was reported to be low (18), Thus, TSA-seq requires a large number of cells (>10 million) to obtain sufficient material and is not easily implemented in studies with small numbers of cells. Ultimately, a method that can reliably identify various genomic structures from a lower number of cells is needed as it opens the possibility of mapping from *in vivo* sources where cell numbers are low.

Of these existing methods, TSA-based labeling has a number of other distinct advantages. For example, it allows labeling of essentially any endogenously expressed protein of interest as has been shown for the mapping of both LADs and nuclear speckles (18). The TSA reaction is time efficient, available as an optimized commercial kit used regularly for tissue section staining. TSA-based approaches also leverage paraformaldehyde-fixed cells/tissues, which could help mitigate any potential cellular perturbations associated with processing live cells (27–29). Finally, TSA-seq and cTSA-seq utilizes a ‘ChIP’-like pull-down approach and subsequent downstream processing familiarity of

Based on our experience using the APEX2-ID method to identify chromatin regions at or near the NL by Streptavidin-based pull down of chromatin, we explored the use of a chromatin pull down-based version of TSA-seq, which we call cTSA-seq. The chief difference between the cTSA-seq and TSA-seq method is the precipitation of chromatin in cTSA-seq versus naked DNA in TSA-seq. Protein is more efficiently labeled than DNA by the TSA reaction (18). This increase in efficiency suggests that cTSA-seq would reduce the cell number requirements to map NL-chromatin regions. The cTSA-seq method reported here can map chromatin regions at or near the NL from 50 000 cells without the addition of carriers. It can sensitively identify both LADs and additional NL-proximal regions that are heterochromatic and found in the B compartments. As a proof of principle, the cTSA-seq approach can be used to identify chromatin interactions at or near the reassembling NL in fixed early and later G1 cells. We further note that the early G1 chromatin-NL relationship is similar to those mapped from the oncogene-induced senescence cells (30,31). We further extend the utility of cTSA-seq by mapping chromatin at or near the NL from the fixed-frozen mouse tissue sections at different developmental stages.

## MATERIALS AND METHODS

### Reagents

The Biotin-XX-Tyramide Superboost kit (Invitrogen, USA, #2005939) was used for the TSA reaction. The rabbit anti lamin-B1 antibody (Abcam, USA, #ab16048) and a Streptavidin conjugate to Alexa 488 (1:200, Biolegend, #405235) were used for fluorescence staining.

### Cultured cells and tissues for cTSA-seq

All animal procedures were approved by the Institutional Animal Care and Use Committee at the Carnegie Institution. Wild-type C57BL/6 mice were housed under a 12 h/12 h light/dark cycle and fed *ad libitum*. Brains were fixed 4% paraformaldehyde for 24 h at 4°C. The tissue was cryoprotected with 30% sucrose, embedded in O.C.T. freezing media and sectioned on a Leica CM3050S cryostat to a thickness of 12–50 μm.

Isolation of cells/nuclei from tissue section was done as follows. Serial sagittal brain sections (∼20–50 μm thick) on glass slides were submerged with PBS supplemented with 0.1% Tween-20 and all tissue except the cerebellar region was scraped off using a P200 pipet tip. The slides were then gently washed with PBS in a coplin jar. The cerebellar regions were then isolated in PBS supplemented with 0.1% Tween-20, briefly spun at 12 000g for 30 s and carefully aspirated. The cerebellar tissue was scraped off the glass slides and resuspended in 1.3 ml of RIPA buffer (50 mM Tris, 150 mM NaCl, 0.1% (wt/vol) SDS, 0.5% (wt/vol) sodium deoxycholate and 1% (vol/vol) Triton X-100, pH 7.5) supplemented with phenylmethylsulfonyl fluoride (PMSF) and transferred to a 15 ml conical tube. The tissue suspension was fragmented using a Misonix Sonicator 3000 on wet ice for 2 × 30 s pulses with 30 s of rest in between at a power equal to 6 W. CaCl_2_ was added to a final of 1 mM and Collagenase Type 2 (Worthington #LS004176) to a final of 0.2 mg/ml. The suspension was digested at 37°C for 60 min and the reaction was halted by the addition of EGTA to a final of 2 mM. The liberated cells/nuclei were filtered through 100 μm nylon mesh, pelleted at 2000g × 3 min and washed with PBS + 0.1% Tween-20. We counted the number of cells/nuclei by staining an aliquot of the cell/nuclei with DAPI and then simultaneously imaging both DAPI and bright-field of the hemocytometer grid on an Olympus E800. On average, we obtained around 100 000 cells/nuclei per 100 μm of medial adult (5 months old) cerebellum and 100 000 cells/nuclei per 200 μm of P0.5 cerebellum. The cells/nuclei were immediately used for immunostaining and cTSA-seq as described below or frozen at –80°C prior to ChIP-seq. We note that cell/nuclei isolation from fixed tissue sections can be done with PBS instead of RIPA buffer.

HCT116 (ATCC, #CCL-247), K562 (ATCC, #CCL-243), hTERT-RPE-1 (ATCC #CCL-4000) and mouse embryonic fibroblasts (MEFs) were cultured in McCoy's 5a with 10% FBS (HCT116), IMDM media with 15% (K562), DMEM-F12 with 15% (hTERT-RPE-1), and DMEM supplemented with 15% FBS, respectively. HCT116, hTERT-RPE-1 and MEFs were cultured in 100 or 150 mm dishes. K562 cells were cultured in T25 flasks. All cells were cultured at 37°C in 5% CO_2_. Bulk cell populations were counted using a hemocytometer and experiments with lower cell numbers were performed by serially diluting of the harvested cells.

### Nocodazole block, release and harvest for early and later G1 cells

HCT116 or hTERT-RPE-1 cells were cultured in three 150 mm dishes to ∼40–60% confluency per experiment and then exposed to nocodazole (100 ng/ml) for 18 h. The cells were washed two times with 10 ml of pre-warmed media and mitotic cells were shaken off and seeded onto 100 mm dishes that were pre-coated with poly-lysine. A fraction of the shake-off cells was saved and fixed with 1% paraformaldehyde for 10 min at room temperature to check for purity of the shake-off population by FACS (see below). The seeded cells were harvested at the indicated post-seeding times by trypsinization and then immediately fixed with 1% paraformaldehyde for 10 min at room temperature. For experiments with G1, S and G2 phases, asynchronous cultures were harvested by trypsinization and fixed for 10 min with 1% paraformaldehyde. Paraformaldehyde fixation was neutralized with 125 mM glycine and the cells were pelleted at 300g × 5 min and washed with phosphate buffered saline (PBS).

### Fluorescence activated cell sorting (FACS) to analyze and isolate cells at different stages of cell cycle

All FACS were performed on a BD FACSaria III machine and on average we obtained 800 000 to 1 million cells unless noted otherwise. To isolate asynchronous cell cycle stage-specific populations (e.g. G1, S and G2), and the early or later G1 populations obtained after nocodazole block, cells were fixed as described above and the resuspended in HBSS + 2% FBS + 10 μg/ml Hoechst 33342. The cells were incubated with Hoechst 33342 for at least 20 min at room temperature before FACS. For nocodazole block and release, we rejected samples where the shake-off mitotic population contained >1% of contaminating G1 cells.

For SLAM-seq experiments (see section, ‘Thiol (SH)-Linked Alkylation for the Metabolic sequencing of RNA’ below), FACS based cell isolation was done with live cells stained with 10 μg/ml Hoechst 33342 in the last 20 min of culture. Since FACS was done with live cells, we limited sorting time to a maximum of 10 min in order to reduce the time between 4sU labeling and RNA harvest. Sorting for SLAM-seq experiments yielded between 30 000 and 100 000 cells.

### Chromatin pull down-based TSA-seq (cTSA-seq)

Paraformaldehyde (1%)-fixed cell populations were permeabilized with PBS containing 0.25% Triton X-100 for 10 min at room temperature. Permeabilized cells were pelleted at 200g for 5 min and the supernatant was carefully removed using a P100 pipette. The cells were then resuspended in PBS + 1% hydrogen peroxide and incubated for 20 min at room temperature to neutralize endogenous peroxidases. The suspension was then pelleted at 200g for 5 min and carefully aspirated with a P100 pipette. The cells were blocked for 20 min in PBS containing 10% normal goat serum, 10% BSA and 10 mM sodium azide and pelleted as described above. The cells were resuspended by gently flicking the tube in the same blocking buffer containing 1:250 dilution of rabbit anti lamin-B1 antibody (Abcam, #ab16048) and incubated overnight at 4°C in a microfuge tube rack. The following day, the cells were pelleted at 200g for 5 min and aspirated with a pipette. The pellet was washed 2 times for 15 min each with PBS supplemented with 0.001% Tween-20. The secondary antibody staining and TSA reaction reagents were from a commercial Biotin-XX-Tyramide Superboost kit (Invitrogen, #2005939) and were used as follows. The primary antibody stained cells were resuspended by gently flicking in ∼100 μl of the provided anti-rabbit horseradish peroxidase (HRP) secondary antibody solution and incubated in a microfuge tube rack at room temperature for 2 h with intermittent mixing. The cells were pelleted at 200g for 5 min, washed 3 times for 15 min each with PBS + 0.001% Tween-20 and one time with 1${\rm{\underset{\rm{{\cdot}}}{s}}}$ reaction buffer supplied with the biotin tyramide kit. The reaction was performed as described for the Biotin-XX-Tyramide Superboost kit with only one modification. The reaction was incubated in an Eppendorf Thermomixer set to 25°C with 300 rpm shaking for 10 min. The reaction was neutralized by adding PBS + 10 mM sodium ascorbate, 10 mM Trolox and 10 mM sodium azide to halt HRP activity and pelleted at 200g for 5 min. The cells were then resuspended in PBS + 0.001% Tween-20 by flicking. A small aliquot was taken to confirm the reaction by fluorescence staining (see below) and the remainder was pelleted and stored at −80°C or immediately used for fragmentation and Streptavidin pull down (see below).

### Fluorescence staining for the cTSA-seq reaction

Cells from the completed cTSA-seq reaction were resuspended in PBS containing 0.001% Tween-20 and Streptavidin conjugated to Alexa 488 (1:200, Biolegend, #405235) and an anti-rabbit secondary conjugated (1:1000) to an Alexa 594 fluorophore. The cells were then stained with DAPI and mounted in Prolong anti-fade gold for imaging on a Leica SP5 scanning confocal microscope using the Leica Application Suite v2.7.3.9723. We used a 40× 1.4NA objective with Leica Type F immersion oil. Diffusion of the Streptavidin signal from the lamin-B1 source signal was measured using the lineplot feature in FIJI v2.1.0/1.53c. The iterative Levenberg-Marquardt algorithm (R package minpack.lm v1.2-1) was used to fit the fluorescence signal to the equation: *y* = *y*_0_ + *A* * exp(*R*_0_ * *x*).

### Immunofluorescence staining of tissue section

Tissue sections were permeabilized with PBS + 0.25% Triton X-100 for 20 min at room temperature. Sections were washed with PBS and then blocked with PBS containing 10% BSA, 10% goat serum and 10 mM sodium azide for 1 h at room temperature. The indicated antibodies were diluted in the blocking solution (PBS, 10% normal goat serum (v/v), 10% BSA (w/v), 10 mM sodium azide) and incubated at room temperature for 24–48 h. Secondary staining of primary antibody was performed with Alexa Fluor conjugated antibodies in PBS containing DAPI. The tissue sections were mounted in Prolong anti-fade gold and imaged on a Leica SP5 scanning confocal microscope using the Leica Application Suite v2.7.3.9723. A 40× 1.4NA objective with Leica Type F immersion oil. Images were processed with FIJI/ImageJ v2.1.0/1.53c. The antibodies used are as follows: rabbit anti-Math1/Atoh1 (dilution 1:500), which was a generous gift from Dr Jane Johnson (University of Texas, Southwestern) (32), rabbit anti-Pax6 (Millipore, #AB2237, 1:300), rabbit anti-Gabra6 (Developmental Studies Hybridoma Bank, #Gabra6-R25, 1:250) (33) and chicken anti-Map2 (Abcam, #ab5392, 1:500).

### Streptavidin pull down of chromatin at or near the nuclear lamina

Cultured cells were resuspended in 300 μl RIPA buffer (50 mM Tris, 150 mM NaCl, 0.1% (wt/vol) SDS, 0.5% (wt/vol) sodium deoxycholate and 1% (vol/vol) Triton X-100, pH 7.5) supplemented with phenylmethylsulfonyl fluoride (PMSF) and incubated at 4°C with end-over-end rotation for 30 min. The suspension was sonicated on a Diagenode Bioruptor Pico and a 50 μl aliquot of the resulting lysate was collected for the input while the remainder was used for the pull down portion of the experiment. The input was digested with 10 μl of 10 mg/ml Proteinase K overnight at 50°C with 700 rpm shaking. For the Streptavidin pull down (StrePD), Streptavidin coated magnetic beads (Pierce, #88817) were washed and resuspended in RIPA buffer. We added ∼70 μl suspension of Streptavidin magnetic beads per pull down. The following day, the beads were collected and washed sequentially with two times in 1ml RIPA, one time in 1ml LiCl buffer (250mM LiCl, 1% IGEPAL-CA630, 1% deoxycholic acid, 10 mM Tris, pH 8.0, 1 mM EDTA), one time in 1 ml with high salt buffer (1 M KCl, 50 mM Tris–Cl pH 8.0, 5 mM EDTA), one time in 1 ml urea wash buffer (2 M urea, 10 mM Tris–Cl pH 8.0), and then one time in 1 ml RIPA. The beads were resuspended in 50 μl of RIPA buffer and digested with 5 μl of 10mg/ml Proteinase K overnight at 50°C with 700 rpm shaking. The input and Streptavidin pull down (StrePD) DNA were purified using Ampure XP beads (Agencourt). A step-by-step protocol is provided in the supplemental material.

For cTSA-seq of *in vivo* samples, we resuspended cells/nuclei isolated from tissue sections in 1.3 ml RIPA supplemented with PMSF and transferred the suspension to a 15 ml conical tube. This suspension was sonicated on wet ice with a Misonix Sonicator 3000 using a 30 s on/off regime for 30 min at a power of 6 W. We chose the probe sonicator since the performance, in our hands, on tissue section derived cells/nuclei was more consistent than the Bioruptor Pico. The fragmentation of chromatin after sonication was largely incomplete, and was 10 kb and greater in size. To further fragment the chromatin, the sonicated lysate was supplemented with CaCl_2_ to a final of 1 mM and MNase (Takara, #2910A) was added to a final of 200 U/ml. The mixture was incubated at 37°C for 60 min and the reaction was halted by the addition of EGTA to a final of 2 mM. At this point, a 10% input was taken and digested with Proteinase K overnight at 55°C and the remainder of the lysate was used for Streptavidin pull-down, which was done at room temperature for 1 h. The expected fragmentation (200–1000 bp) of input DNA was verified on an Agilent Bioanalyzer and samples were rejected if fragmentation was poor. We also excluded samples where MNase treatments resulted in excessive mono-nucleosomes due to significant loss of genome coverage of the input DNA.

### TSA-seq

The TSA reaction was performed in a manner similar to cTSA-seq with the same rabbit polyclonal lamin-B1 (Abcam, #ab16048) antibody. We used 200 million K562 cells for each replicate and the TSA reactions were confirmed by immunofluorescence as described above. Cells were treated with Proteinase K overnight at 55°C, and DNA was isolated by the phenol/chloroform/isoamyl alcohol method. The biotinylation of DNA was examined by dot blotting onto positively charged nylon membranes (Thermo, BrightStar), drying overnight and then probing with Streptavidin HRP. We performed the isolation of biotinylated DNA in a manner similar to the ‘later modified protocol’ described by Chen, *et al.*, for TSA-seq (18). Briefly, DNA was sheared with a Misonix Sonicator 3000 to a size between 300–500 bp and 1/10th was set aside as the input. DNA was mixed with a 2× binding buffer (10 mM Tris–Cl pH 7.4, 1 mM EDTA, 2 M NaCl) and biotinylated DNA was enriched with Streptavidin MyOne C1 beads using end-over-end rotation for 1 h at room temperature followed by 8 h at 4°C. Washing of Streptavidin beads was done using 5× changes of 1× binding buffer supplemented with 0.05% Triton X-100. Each wash consisted of an initial incubation at 55°C for 2 min followed by shaking (900 rpm) at the same temperature for an additional 2 min. The streptavidin beads were transferred to a new tube after each wash. The DNA was released from the Streptavidin beads by overnight digestion with Proteinase K and DNA was isolated using Ampure XP beads.

### Thiol (SH)-linked alkylation for the metabolic sequencing of RNA (SLAM-seq)

To determine new transcription for FACS isolated unsynchronized G1 (from asynchronous cell culture) or synchronized early and later G1 cells, we incubated asynchronous HCT116 cultures or re-seeded mitotic shake-offs of HCT116 cells with 500 μM 4-thiouridine (4sU) for 1 h prior to harvest. Twenty minutes before harvest, we pre-stained cellular DNA by directly adding Hoechst 33342 to a final of 10 μg/ml. The cells were harvested by trypsinization, pelleted at 150g for 3 min each and resuspended in HBSS + 2% FBS supplemented with 10 μg/ml Hoechst 33342 that was pre-warmed to 37°C. The cells were immediately filtered with a 0.45 micron filter and subject to FACS as described above with the exception that the cell sorting chamber was also set to 37°C since the cells were alive. FACS was performed for a maximum of 10 min, which usually yielded anywhere between 30 000 and 100 000 cells. The collected cells were then pelleted at 500g for 3 min, aspirated with a P100 pipette and RNA was immediately extracted using the Qiagen RNeasy Plus kit and quantitated. To convert the incorporated 4sU, we incubated the RNA (∼0.5–1 μg) in PBS pH 7.4 with 100 μM iodoacetamide at 50°C for 15 min and then re-purified the RNA using the Qiagen RNeasy Plus kit. As a control, we performed iodoacetamide treatment and sequencing on unlabeled (no 4sU treatment) asynchronous G1 cells to provide an estimation of sequencing errors.

### Sequencing library production

RNA library building was done using the Illumina TruSeq RNA library kit v2 with Ribo-depletion. DNA libraries were prepared using the Rubicon Genomics ThruPlex kit. Sequencing was performed on the Illumina NextSeq 500 platform.

### Data processing

Mapping of raw reads was done using the hg19 or mm10 assemblies and Bowtie 2.3.2 under the default setting. Duplicates were removed using samtools v1.6. The reads were then called into 10 or 100 kb genomic windows using the coverage function in bedtools v2.26.0. Next, we normalized the StrePD and input read count data to one million, calculated the log_2_(StrePD/input) value and transformed the data to a *z*-score. The publicly available LADs and lamin-B1 ChIPseq datasets examined in this study were analyzed as described above. The depmixS4 v1.4-0 R package was used to call a three-state Hidden Markov Model (HMM) to identify LADs coordinates. The three-state model parsed the cTSA-seq signal into regions that contained (i) clear NL-chromatin signal enrichment, (ii) intermediate or noisy signal and (iii) clear depletion. We intersected the HMM calls between experimental replicates to find LAD regions that were reproducibly identified. Quantitation of epigenetic and lamin-B1 signals was done using the bigwigAverageOverBed function from the Kentutils v3.62 package. For analyses that examined chromosomes as a percentage of their length, we first split chromosomes in 200 windows using the makewindows function in bedtools and then quantitated the lamin-B1 signal over these windows using the bigwigAverageOverBed function from Kentutils. A/B compartment scores were calculated using CscoreTool v1.1 (34). Analysis of sequencing depth was done with Preseq v2.0.3. Additional analysis, statistics and graphical plotting were done either in R Studio v0.98.95.3 using the pHeatmap v1.0.12, corrplot v0.84 and Hmisc v4.2-0 packages or Microsoft Excel 2016. Browser tracks were displayed using the UCSC Genome Browser with a smoothing window of 2.

For SLAM-seq experiments reads were initially trimmed by 10 nucleotides from both the 5’ and 3’ ends using cutadapt v1.6. The trimmed RNA-seq data was aligned with Bowtie 2.3.2 and Tophat2 under default settings using the Ensembl hs75 assembly. Polymorphisms corresponding to the expected U- to C-conversions were quantitated using the GrandSLAM v 2.0.5c package (35). Data was filtered for a minimum of 10 transcripts per million (TPM) and the new to total RNA (NTR, ‘MAP’) value was used to represent the gene for subsequent analysis. Statistics and graphical plotting were done either in R Studio v0.98.95.3 using base functions, pHeatmap v1.0.12, corrplot v0.84, car v3.0-5 and Hmisc v4.2-0 packages, or Microsoft Excel 2016.

### Statistical analyses

**Figure 1.**
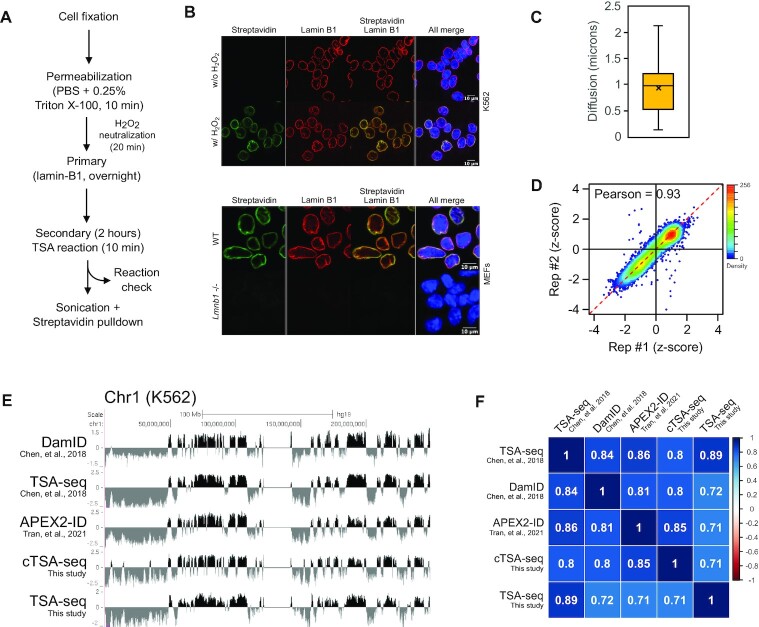
(**A**) The cTSA-seq experimental pipeline used in this study. (**B**) The lamin-B1 cTSA-seq reaction done with or without hydrogen peroxide using K562 cells (top panel) and with hydrogen peroxide in control (‘WT’) and *Lmnb1*–/– MEFs (bottom panel). Staining was done with Streptavidin (green) to highlight biotinylated material and a secondary antibody toward the lamin-B1 antibody (red). Scale bar equals 10 microns. (**C**) Boxplot showing the observed distance of diffusion (in microns) of the cTSA-seq labeling reaction from the lamin-B1 source. (**D**) Scatter plot showing the relationship between the cTSA-seq replicates. The Pearson coefficient is 0.93. (**E**) UCSC Genome browser view of lamin-B1 cTSA-seq from K562 cells (Chr1, hg19). DamID (Chen *et al.*, 2018), TSA-seq (Chen, *et al.*, 2018; lamin-B clone 2D8), APEX2-ID (Tran *et al.*, 2021), cTSA-seq (This study; lamin-B1, ab16048), and TSA-seq (This study; lamin-B1, ab16048) mapping profiles are shown for comparison. The y-axis represents an averaged z-score for each dataset. (**F**) Pearson correlation matrix for DamID (Chen *et al.*, 2018), TSA-seq (Chen *et al.*, 2018), APEX2-ID (Tran *et al.*, 2021), cTSA-seq (This study, lamin-B1, ab16048), and TSA-seq (This study, lamin-B1, ab16048) mapped at 100 kb resolution.

Pearson correlations seen in Figures 1D, F, 3B and Supplementary Figure S2D were calculated using the rcorr(x, method = ‘pearson’) function from the Hmisc v4.2-0 package or the cor.test function in base R. Two-sided *t*-tests calculated for Figures 4D and Supplementary Figure S4E were calculated using the base R function t.test().

### Data availability/sequence data resources

**Figure 2.**
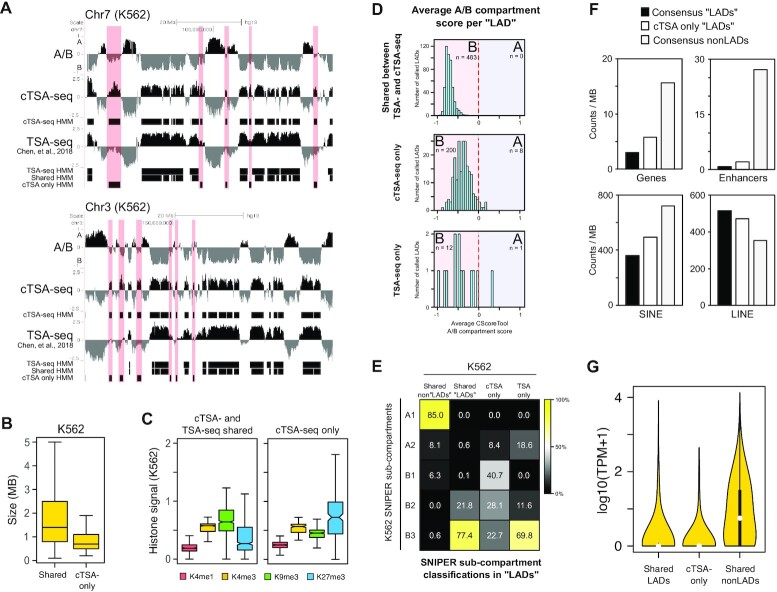
(**A**) Example UCSC Genome Browser tracks for select regions of chromosomes 7 and 3 (hg19). The K562 Hi-C dataset is presented as an A/B compartment track (‘A/B’) where the A compartment is represented by positive values and the B compartment by negative values. cTSA-seq and TSA-seq (Chen *et al.*, 2018; lamin-B, clone 2D8) data is presented as an averaged *z*-score. The three-state HMM tracks are shown below each track. The ‘Shared HMM’ track represents an intersection between cTSA-seq and TSA-seq HMM calls. The light red bars highlight regions that were called by the HMM as strong signal for cTSA-seq only and coincides with the ‘cTSA-seq only HMM’ tracks. (**B**) Boxplot showing the size of cTSA-seq and TSA-seq (‘Shared’) NL-chromatin regions and cTSA-seq only (‘cTSA- only’) regions. (**C**) Boxplots showing the ENCODE histone modification signals measured in ‘cTSA- and TSA-seq shared’ NL-chromatin regions (left) and ‘cTSA-seq only’ regions (right). H3K4me1 is shown in red, H3K4me3 in yellow, H3K9me3 in green and H3K27me3 in blue. (**D**) Frequency histograms showing the number of called chromatin regions at or near NL, referred to as ‘LADs’, and their average Hi-C A/B compartment score. The A/B compartment score was calculated from K562 Hi-C data using the CscoreTool program and these scores were averaged over the identified NL-chromatin region coordinates. The top panel pertains to the regions ‘Shared between cTSA- and TSA-seq’, the middle panel pertains to ‘cTSA-seq only’ regions and the bottom panel pertains to TSA-seq only regions. The B-compartment is shaded light red and the A compartment is shaded blue. The number of called ‘LADs’ falling into each compartment is noted beneath the ‘A’ or ‘B’ label for each compartment. (**E**) Heatmap showing the percentage of SNIPER-defined K562 Hi-C sub-compartments in K562 shared cTSA- and TSA-seq (‘Shared LADs’), cTSA-seq only (‘cTSA-only LADs’) and TSA-seq only chromatin regions (‘TSA-only LADs’). Shared nonLADs refers to euchromatic regions identified by both methods. (**F**) Bar charts showing the number of genes (upper left), enhancers (upper right), SINE elements (lower left) and LINE elements (lower right) in LADs shared between cTSA- and TSA-seq (‘Shared LADs’), cTSA-seq only LADs (‘cTSA-only’) and in ‘Shared nonLADs’ regions. (**G**) Violin plot showing the expression level of genes found in ‘Shared LADs’, cTSA-seq only ‘LADs’ (‘cTSA- only’) and in ‘Shared nonLADs’ regions.

Sequencing data generated for this study is deposited at NCBI GEO Accession number GSE186503. K562 cell DamID and TSA-seq LADs data (18) was obtained from Gene Expression Omnibus (GEO) GSE66019. The K562 cell APEX2-ID data (8) was from GEO GSE159482. K562 ENCODE RNA-seq data was obtained from GEO GSM958731. The K562 ENCODE ChIPseq data was obtained from GEO GSM733776. The Tig3 OIS data (30) was obtained from GEO SE76605 and the IMR90 OIS data (31) was obtained from GEO GSE49341. The epigenome data for HCT116 cells was obtained from the ENCODE project (HCT116 reference epigenome series ENCSR361KMF). The HCT116 ENCODE RNAseq data set was obtained from GEO GSM958749. Mouse cell line lamin-B1 DamID data were obtained from the UCSC Genome Table browser (5,36). FACS-isolated adult mouse granule cell H3K9me3 and ATAC-seq data were obtained from GSE95622 and GSE101918, respectively (37,38). SINE and LINE elements were obtained from the UCSC Table Browser, ‘Table: rmsk’, repClass: ‘SINE’ or ‘LINE’. The K562 enhancer data was obtained from http://www.enhanceratlas.org (39). The K562 Hi-C dataset (4) was obtained from GEO GSE63525, and the SNIPER K562 subcompartment track was obtained from https://cmu.app.box.com/s/n4jh3utmitzl88264s8bzsfcjhqnhaa0/folder/86847304302 (40). UCSC Genome browser tracks are available at the following URLs:

Figures 1E, 2A and Supplementary Figure S1D: https://genome.ucsc.edu/s/tranjoseph/hg19_K562_Figures_1%262.

**Figure 3.**
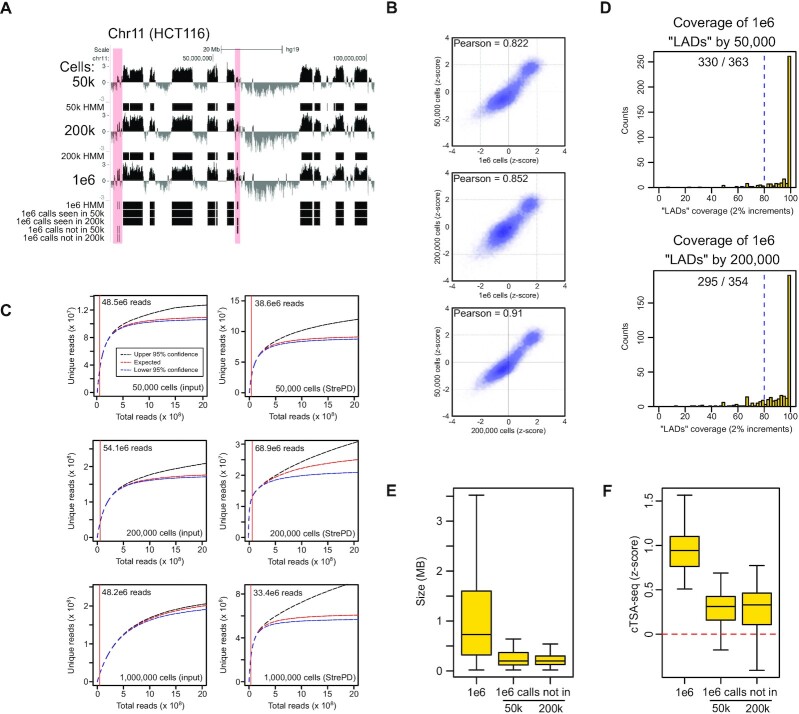
(**A**) UCSC Genome Browser tracks for low cell cTSA-seq (Chr11, hg19). ‘50k’, ‘200k’ and ‘1e6’ represent the 50 000, 200 000 and 1 000 000 HCT116 cells, respectively. Three-state HMM tracks, corresponding to the strongest signals, are shown beneath each signal track. 1e6 HMM calls identified (‘1e6 calls seen in 50k’ and ‘1e6 calls seen in 200k’) and not identified (‘1e6 calls not in 50k’ and ‘1e6 calls not in 200k’) by low cell experiments are shown at the bottom. Light red bars highlight regions from the one million cell sample not called or called as weak signal by the HMM using data from low cell experiments. (**B**) Scatter plots comparing data (*z*-score) from 50 000, 200 000 and 1 000, 000 cell cTSA-seq experiments. Pearson correlation coefficient is shown on the upper left of each plot. (**C**) Representative analysis of sequencing depth and the predicted number of unique reads for each sample from the 50 000, 200 000 and 1 000 000 cell cTSA-seq. The red vertical line indicates the actual sequencing depth for each sample. The read numbers are shown labeled on the upper left. Input samples are the left three plots while Streptavidin pull down (‘StrePD’) samples are on the right and these are denoted on the lower right. (**D**) The coverage of HMM-defined ‘LADs’ regions from the one million cell dataset by those obtained from the low-cell experiments. The coverage of the one million cell by the 50,000 cell data is the top graph while the coverage by 200 000 cells is the bottom graph. The data is presented as a histogram where the y-axis is the number of ‘LADs’ presented as ‘counts’ and the x-axis is the percentage of the ‘LAD’ covered. The coverage bins are in 2% increments. The blue dashed line is the 80% coverage position. The actual number of ‘LADs’ with 80% or greater coverage over the total number of LADs is shown in the middle top part of each graph. (**E**) Boxplot showing the size (in megabases) of the mapped chromatin regions from 1 000 000 cells, and those from the 1 000 000 cell experiment not called by the lower cell number experiments. (**F**) Boxplot showing the cTSA-seq (*z*-score) signal of HMM-defined chromatin regions from 1 000 000 cells, and those from the 1 000 000 cell experiment that were not called in the lower cell number experiments.

Figure 3A and Supplementary Figure S1N: https://genome.ucsc.edu/s/tranjoseph/hg19_low_cell_HCT116_cTSAseq_Figure_3.

Figure 5A, C, F, Supplementary Figures S4A, S4C: https://genome.ucsc.edu/s/tranjoseph/hg19_R90_R180_G1SG2_Lenain_Sadaie_RPE_FIgure_5%26S4.

Supplementary Figure S3B: https://genome.ucsc.edu/s/tranjoseph/hg19_R90_R180_Async_G1_rank_check_Figure_S3.

Figure 6H: https://genome.ucsc.edu/s/tranjoseph/mm10_B6_cerebellum_Reps1_2.

Figure 7: https://genome.ucsc.edu/s/tranjoseph/mm10_B6_TS_P0.5_5m_cTSAseq.

## RESULTS AND DISCUSSION

### Development of chromatin pull down-based Tyramide Signal Amplification-sequencing (cTSA-seq) to reliably map chromatin regions at or near the NL

The Tyramide Signal Amplification (TSA)-seq method utilizes the small fraction of DNA that is labeled by the TSA reaction to map LADs (18). However, the TSA labeling occurs on both DNA and proteins (18,23) that are at or near the nuclear lamina. We first examined the Tyramide Signal Amplification (TSA) reaction with a commercial kit (see Materials and Methods) using K562, HCT116 and mouse embryonic fibroblast (MEF) cell lines and a commercially available rabbit lamin-B1 antibody (Abcam, #ab16048) to target the proximity labeling of proteins at or near the NL with biotin (Figure 1A). The post-reaction staining with an Alexa-conjugated Streptavidin coincided largely with lamin-B1 while Streptavidin staining was not evident in control reactions that omitted the hydrogen peroxide (Figure 1B, top panel), used an anti-mouse HRP secondary antibody (Supplementary Figure S1A) or used lamin-B1 null MEFs (Figure 1B, bottom panel). Similar to previous work (18), the diffusion of Streptavidin signal for our unmodified TSA reaction was ∼1 μm from the primary lamin-B1 antibody label (Figure 1C).

Next, we sonicated and pulled down the fragmented biotinylated chromatin (Supplementary Figure S1B) with Streptavidin beads for sequencing (Figure 1A). We used one million K562 cells, which is at least 10 times less than that required for TSA-seq (41). The data obtained from cTSA-seq biological replicates were highly correlated (Figure 1D, Pearson = 0.93). The average cTSA-seq signal was similar to those obtained by DamID, the original published TSA-seq method using the mouse monoclonal lamin-B 2D8 clone antibody (18), our TSA-seq using the rabbit lamin-B1 antibody (ab16048) for this study, and our recently adapted APEX2-ID method (Figure 1E, F) (8). To further assess the cTSA-seq data, we performed decile analysis as done for SON TSA-seq (18). The published decile analysis for SON TSA-seq data revealed a gradient of epigenetic signal and transcription activity across the nuclear speckle-lamin TSA-seq axis (18), with elevated levels of transcriptional activity and euchromatic marks closest to the nuclear speckle. Conversely, these signatures were weakest at the NL. We stratified the lamin-B1 cTSA-seq signal into decile ranks where the 1st and 10th ranks corresponded to the lowest and highest cTSA-seq log_2_ values, respectively (Supplementary Figures S1C, S1D); and then measured the TSA-seq, epigenetic and transcriptional signal within genomic regions defined by each decile. Similar to what was observed for TSA-seq, cTSA-seq deciles showed an increasing gradient of transcriptional activity and euchromatic epigenetic signal away from the NL, which is represented by the lower deciles (Supplementary Figure S1E, top four panels). As anticipated, elevated heterochromatic signatures were closest to the NL (Supplementary Figure S1E, bottom two panels).

We next used a three-state Hidden Markov Model (HMM) to identify NL-chromatin regions and then intersected the calls from each replicate to identify the strongest signals that were also reproducible. This approach was previously used to identify NL-associated chromatin mapped by APEX2-ID (8). The three-state HMM model used here separates the cTSA-seq data into (i) strong signal representing clear enrichment, (ii) intermediate or noisy signal and (iii) signal that is not enriched by the procedure. The performance of the three-state HMM model is comparable to the binary two-state model at the 100 kb resolution, and results in a similar number of NL-chromatin region calls and feature sizes (Supplementary Figures S1F, G). The three-state HMM calls at 100 kb contain the highest decile ranking signal (Supplementary Figure S1D, compare ‘cTSA-seq HMM’ and ranks 7–10 tracks). We found that the cTSA-seq approach identified 97.3% of the LADs identified by the same three-state HMM model from data generated by the related published TSA-seq method (18). The genome coverage of NL-chromatin regions was approximately 1.2GB, or around 39.7% of the genome, and was ∼158 to 178MB greater than the genomic coverages observed for DamID or TSA-seq methods, respectively (Supplementary Figure S1H).

The three-state HMM identified cTSA-seq-specific enrichments (*n* = 208) that were not identified in the published TSA-seq data (Figure 2A, light red bars). To verify this, we performed TSA-seq using the same antibody we used for cTSA-seq and compared it with our cTSA-seq data. We found that the majority (75.5%) of the cTSA-seq only regions found were the same between the two cTSA-seq and TSA-seq comparisons. These cTSA-seq-specific chromatin showed weak TSA-seq signal (Supplementary Figure S1I). These cTSA-seq specific regions were generally smaller in size (Figure 2B, <1MB), enriched for H3K27me3 (Figure 2C) and contained weaker cTSA-seq signal compared to shared NL-chromatin regions (Supplementary Figure S1J). The cTSA-seq only regions covered around 6.02% of the genome (Supplementary Figure S1H).

To gain further understanding of the cTSA-seq specific regions, we examined the Hi-C compartments associated with these chromatin regions. Previous analyses of Hi-C data have broadly identified the A and B compartments, which correspond to open euchromatin and closed heterochromatin, respectively (3,4). We calculated the A/B Hi-C compartment score for K562 cells at a 20 kb resolution using the CscoreTool program developed in our lab, and then measured the average A/B compartment score within the defined NL-chromatin regions (4,34). The NL-chromatin regions identified by DamID, TSA-seq and cTSA-seq methods all showed an enrichment for B-compartment scores (Supplementary Figure S1K) and as anticipated, all chromatin regions identified by both lamin-B TSA- and lamin-B1 cTSA-seq methods (‘shared’ regions) were classified as being in the B-compartment (Figure 2D, top panel). Most (96.2%, 200 out of 208) of the cTSA-seq only chromatin regions were also classified as the B-compartment (Figure 2D, middle panel). To further explore this observation, we compared our cTSA-seq data with the published SNIPER K562 sub-compartment track (4,40). The SNIPER algorithm further subdivides the A and B compartments into A1-2 and B1-4 sub-compartments at 100 kb resolution, respectively, but excludes the B4 sub-compartment since this subcompartment was only found on chromosome 19 (40). NL-chromatin regions mapped by DamID, TSA-seq and cTSA-seq all showed the expected enrichment for SNIPER B sub-compartments (Supplementary Figure S1L). An intersection of TSA- and cTSA-seq shared chromatin regions with the SNIPER sub-compartment data revealed enrichment for B3 and B2 compartments (99.2%, Figure 2E) while cTSA-seq specific regions contain signatures for B1, B2 and B3 compartments (91.5%), with a notable bias toward the B1 compartment (Figure 2E). Our three-state HMM analysis at 100 kb also identified a small number (*n* = 13) of TSA-seq only regions that were classified largely as being in the B-compartment based on their average compartment score (Figure 2D, bottom panel), and contained an enrichment for SNIPER B sub-compartments (Figure 2E). However, we did not further examine these due to their low number. We also found that cTSA-seq only regions were more enriched for genes, enhancers, SINE elements and contained lower LINE element content than TSA- and cTSA-seq shared ‘LADs’ regions (Figure 2F). Both the shared and cTSA-seq specific regions have low gene expression (Figure 2G). These analyses show the cTSA-seq method can be used to map chromatin in the B-compartment that includes previously defined LADs and heterochromatin regions that are near the NL.

The identification of chromatin regions found in the B-compartment but not in the previously defined LADs is interesting as it suggests that the B-compartment defined by Hi-C contains both LADs and chromatin regions near LADs. The ease of biotinylation of proteins near the NL by diffusion may lead to the pull down of chromatin proximal to the NL that is in a closed chromatin state. Additionally, the chromatin based pull down by the cTSA-seq method may capture both labeled naked DNA and chromatinized DNA thereby increasing mapping sensitivity.

### cTSA-seq mapping of chromatin at or near the NL in as few as 50 000 cells without adding carriers

The efficient mapping of chromatin regions in LADs or near the NL from low numbers of cells would open up the possibility of characterizing rare cell populations directly isolated from *in vivo* sources without further culturing or from subpopulations of cells sorted from *in vitro* culture. LADs mapping has been reported for low-cell populations using DamID and CUT and RUN (GO-CaRT) methods (16,22,42) but was not applicable to the original TSA-seq method.

We therefore examined the possible use of cTSA-seq to map chromatin regions at or near the NL in a lower number of cells. We titrated HCT116 cells down to 50 000 and 200 000 cells prior to performing immunostaining with lamin-B1 and cTSA-seq. A one-tenth input was collected from the sonicated lysate prior to Streptavidin pull down. The lamin-B1 cTSA-seq profiles from the 50 000 and 200 000 cell samples were visually similar to those mapped from one million cells (Figure 3A) and well correlated with the 1 000 000 cell cTSA-seq (Pearson > 0.82, Figure 3B) and our previously published APEX2-ID dataset (Supplementary Figure S1M) using 1 million cells (8). We did observe that cTSA-seq experiments with very low numbers of cells (e.g. 100 to 20 000 cells) often suffered from inconsistency and high noise (Supplementary Figure S1N). Based on the unique and total reads, our sequencing depth did not reach saturation (Figure 3C) and we were able to achieve good mapping from as few as 38.6 million reads.

We next applied the same three-state HMM model used above to our low-cell data to call the strongest reproducible chromatin regions at or near the NL (Figure 3A, ‘HMM’ tracks). Intersecting these HMM calls revealed that the low-cell cTSA-seq can identify the majority of NL-chromatin regions (>82%) found in the 1 million cell reference sample. Most of these 1 million cells reference ‘LADs’ also identified by the low cell data (84–91%) showed 80% or greater coverage by the low cell mapping (Figure 3D), and indicates that we can achieve reasonably complete mapping from 50 000 cells. We did notice that smaller regions with an average size of ∼250 kb were less efficiently identified by the HMM model when using data from low-cell cTSA-seq experiments (Figure 3A, light red boxes, Figure 3E). However, these regions missed by the HMM calls had relatively weak signals in all samples (Figure 3F) suggesting that these missed calls were filtered out by the three-state HMM model.

We show that cTSA-seq can offer high quality mapping of chromatin at or near the NL in as few as 50 000 cells without adding any carriers. We have previously developed a carrier approach to reduce sample loss in low-cell ChIP-seq, and have successfully used this approach to map epigenetic marks and transcription factor binding sites from as few as 100 cells (43,44). Adapting this carrier approach to be compatible with cTSA-seq should enable mapping of chromatin regions at or near the NL in far fewer cells than 50 000, especially considering that these chromatin regions represents 30–50% of the genome. Overall, the data presented here shows that cTSA-seq is a complementary tool that can efficiently identify chromatin regions consisting of dominantly LADs and NL-proximal chromatin found in the B compartment from as few as 50 000 cells.

### cTSA-seq enables the use of fixed and sorted cells to more accurately identify chromatin regions at or near the assembling NL as cells progress through G1 phase

A recent study on the dynamics of LADs during the cell cycle revealed an increased interaction between the distal ends of chromosomes and the reassembling NL during early G1 in synchronized cell populations (7). This study collected all cells at a fixed time point after mitotic block and release for mapping. However, mitotic block does not arrest all cells in mitosis and the arrested cells do not enter interphase with perfect synchrony after release (Supplementary Figure S2A). Therefore, mapping using all released cells would affect the results. Since cTSA-seq offers high quality mapping of only 50 000 cells, we reasoned that we should be able to improve the mapping by adding a sorting step for G1 DNA content of fixed cells to exclude contaminating un-arrested cells or cells that have not entered into G1.

**Figure 4.**
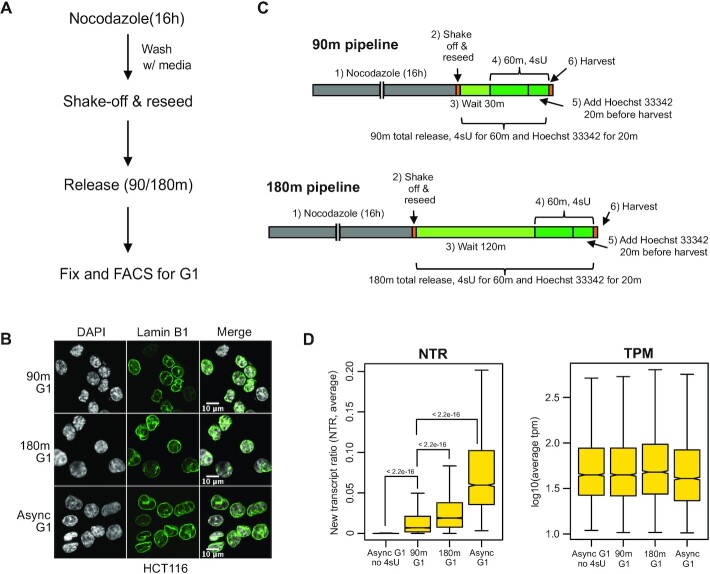
(**A**) Illustration of experimental workflow for nocodazole block and release protocol. (**B**) Lamin-B1 immunostaining (green) for HCT116 cells in early G1 (‘90m G1’), later G1 (‘180m G1’) and asynchronous G1 (‘Async G1’) isolated from asynchronous cell cultures. DAPI is presented in gray and lamin-B1 is presented in green. The scale bar equals 10 microns. (**C**) Experimental pipeline for performing SLAM-seq experiments on early (‘90m pipeline’) and later (‘180m pipeline’) G1 HCT116 cells. The 4sU labeling time was kept constant at 60 min for each sample. (**D**) Boxplots showing the average new transcripts to all transcripts ratio (NTR, left) and a log_10_ transformation of the transcripts per million (TPM, right) for indicated samples. The *P*-values shown in the boxplot were calculated from a two-tailed t-test.

We performed nocodazole arrest of HCT116 cells followed by a mitotic shake-off and release into fresh media followed by fixation (Figure 4A). Fluorescence Activated Cell Sorting (FACS) analyses showed that some mitotic HCT116 cells released from nocodazole progressed into G1 of the cell cycle (Supplementary Figure S2A, left panel). A clear but minor G1 cell peak could be seen between 60–90 min after release, and by 180–240 min the profile was similar to that seen for asynchronous cultures suggesting that much of the cell population had finished mitosis (Supplementary Figure S2A, left panel). We next used this approach to FACS isolate the fixed G1 cell population at 90 or 180 min after mitotic release. These cell populations will be referred to as ‘90m G1’ or ‘180m G1’ cells, respectively, to differentiate them from the G1 population isolated from asynchronous cell populations (‘Async G1’). We found that both 90m and 180m G1 cells have entered G1 as judged by DAPI and lamin-B1 staining (Figure 4B). Importantly, lamin-B1 staining appeared strongly at the nuclear periphery thus allowing the use of cTSA-seq. Chromatin in many 90m G1 nuclei appeared noticeably dissimilar to 180m G1 and asynchronously growing G1 HCT116 cells (Supplementary Figure S2B) as has been previously observed for synchronized cells released from mitotic block (45). Using the same nocodazole block and release protocol, we similarly isolated early (90m) or later (180m) G1 hTERT-RPE1 cells. Based on the FACS profile, hTERT-RPE1 cells progressed into G1 slower (7) than that of HCT116 cells (Supplementary Figure S2A, right panels), but similar to HCT116 cells, staining revealed that cells have entered G1 at both time points (Supplementary Figure S2C).

Next, we further confirmed that the 90m and 180m G1 cells described above represented the early and later stages of G1, respectively. Previous reports indicate that transcription progressively increases in post-mitotic G1 (46,47). To accomplish this, we used the Thiol (SH)-Linked Alkylation for the Metabolic sequencing of RNA (SLAM-seq) labeling strategy (Figure 4C) to measure new transcripts (48). Cells treated with 4-thiouridine (4sU) incorporate the analog into newly transcribed RNA (49), and treatment of RNA with iodoacetamide results in a U- to C-conversion of the 4sU analog during reverse transcription that can be quantitated and expressed as a ratio of new RNA transcripts to total RNA (NTR) (48,50). We performed SLAM-seq on 90m G1, 180m G1 and asynchronous G1 samples by labeling with 4sU for 60 min prior to the harvesting time point (Figure 4C). The replicate experiments were concordant (Supplementary Figure S2D), and a low level of new transcription was observed in 90m G1 cells that further increased in the 180m G1 cells (Figure 4D, left panel, *P*-value < 2.2e^−16^) even though global transcript levels remained similar (Figure 4D, right panel). The expression of select cell cycle related (e.g. *CDT1*, *CDK4*, *CDK6* and *PCNA*) and DNA replication related genes (e.g. *ORC1*, *ORC6*, *MCM3,7,8*) were not prominent in 90m G1 samples, but was more apparent in 180m G1 and asynchronous G1 samples (Supplementary Figure S2E). These results are consistent with previous observations and show that the 90m G1 fraction contained cells that has entered early G1 phase characterized by low levels of transcription, while the 180m G1 cells represent a later stage of G1 with an increased number of genes being expressed.

**Figure 5.**
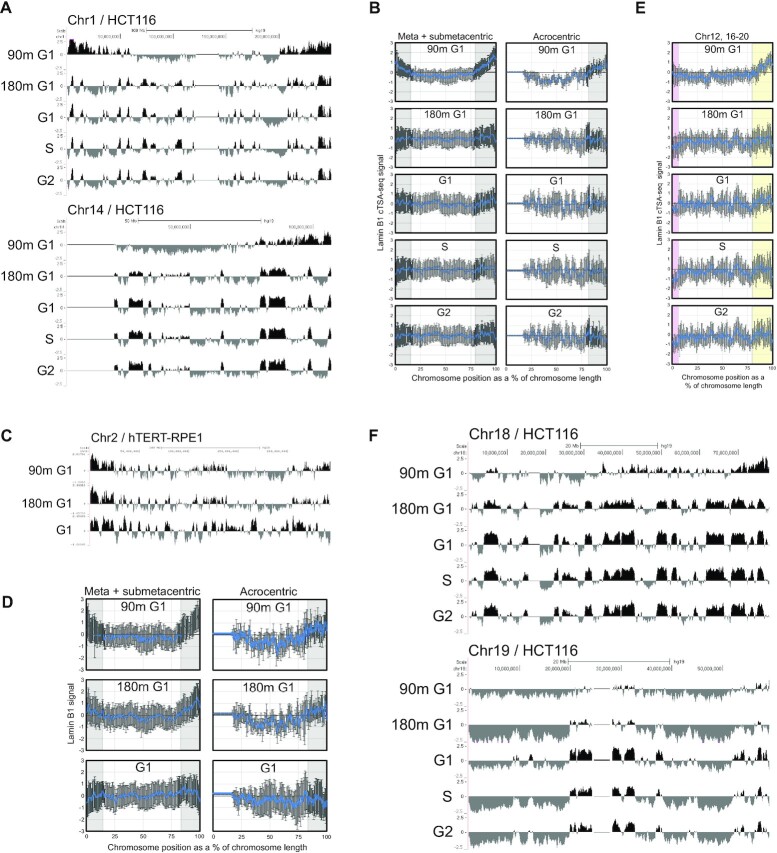
(**A**) UCSC Genome browser tracks (hg19) showing the signal obtained by lamin-B1 cTSA-seq from early G1 (‘90m G1’), later G1 (‘180m G1’), asynchronous G1 (‘G1’), S and G2 sorted HCT116 cells. The metacentric chromosome 1 (top) and acrocentric chromosome 14 (bottom) are displayed. The data presented are average *z*-scores of both replicate experiments. (**B**) Line plots showing the average lamin-B1 cTSA-seq signal from meta- and submetacentric (left) and acrocentric (right) chromosomes in the indicated cell cycle stages. Each chromosome was segmented as a percentage of their length and the lamin-B1 signal was quantified over these windows. Error bars represent standard deviation of signal. Grey bars highlight distal regions of the chromosome that show increased cTSA-seq signal in early G1. (**C**) UCSC Genome Browser tracks (Chr2 2, hg19) mapped by lamin-B1 cTSA-seq from ‘90m G1’, ‘180m G1’ and asynchronous G1 (‘G1’) populations of hTERT-RPE1 cells. (**D**) Average lamin-B1 cTSA-seq signal from meta- and submetacentric (left) and acrocentric (right) hTERT-RPE1 chromosomes. Each chromosome was evenly segmented as a percentage of their length and the cTSA-seq signal was quantified over these windows. The error bars represent standard deviation. Grey bars highlight regions where cTSA-seq signal is enriched during early G1. (**E**) The average lamin-B1 cTSA-seq signal for smaller (chromosomes 12, 16–20) meta- and submetacentric chromosomes. Purple bars highlight the low cTSA-seq signal seen on the short arm of these chromosomes that further decreases in the other samples. The yellow bars highlight the stronger distal cTSA-seq signal seen in early G1. (**F**) UCSC genome browser tracks of lamin-B1 cTSA-seq signal for chromosomes 18 (top) and 19 (bottom) across different stages of the cell cycle. The data presented are average *z*-scores.

We next used cTSA-seq to identify chromatin regions at or near the reassembling NL in early and later G1 phase HCT116 cells. We also performed cTSA-seq of asynchronous G1, S and G2 cells isolated by FACS, which were previously shown to be largely similar with minor variations (7,8). The G2/M population was referred to as ‘G2’ since the nuclear envelope is disassembled during mitosis and does not contribute much to the signal (16,51). Lamin-B1 cTSA-seq mapping revealed a graded enrichment of signals toward chromosome ends in 90m G1 HCT116 cells, suggesting that the reassembling NL is closest to these chromosomal regions (Figure 5A, B grey bars). cTSA-seq mapping of the later 180m G1 population revealed a reduction of signal at chromosome ends and the appearance of signal at chromatin regions that were mapped from asynchronous G1, S and G2 populations (Figure 5A, B, Pearson > 0.7). We also performed the same experiment with hTERT-RPE1 and found a similar enrichment of signal toward the distal ends of chromosomes at the 90m early (Figure 5C, D). Our lamin-B1 cTSA-seq mapping pattern of the early 90m G1 cells is similar to the early NL-chromatin interactions previously observed using an antibody-targeted Dam-ID approach (7). Thus, the cTSA-seq method readily identifies chromatin at or near the reassembling NL in early G1 from FACS sorted cell populations.

In general, the distal enrichment of lamin-B1 signal revealed by cTSA-seq in early G1 cells occurred across metacentric, sub-metacentric and acrocentric chromosomes (Figure 5B, grey bars). It is notable that smaller, non-acrocentric chromosomes, such as chromosomes 12,16–20, had early G1 cTSA-seq signal that was mainly found only on the long (q) arm of the chromosome (Figure 5E, yellow bars) while the short (p) arm of these chromosomes, which showed little or no initial lamin-B1 chromatin signal, underwent a further reduction of the signal in later G1 and asynchronous G1 cells (Figure 5E, purple bars). The well-studied human chromosomes 18 and 19 are known to preferentially localize to the nuclear periphery and nucleoplasm, respectively, and their differential localization was reported to occur in early G1 (13). We found that in early G1, chromosome 18 shows an accumulation of lamin-B1 cTSA-seq signal at one end of the chromosome while chromosome 19 shows little to no distal signal (Figure 5F).

We examined for the possibility of any transcriptional effect due to these NL-chromatin dynamics. To accomplish this, we first defined NL-chromatin regions by the three-state HMM model, as done for the K562 cTSA-seq data described above. The HMM model identified regions that were based largely on signal strength and it identified similar regions as those defined by decile ranking of cTSA-seq signal (Supplementary Figures S3A, S3B). The number of genes (∼5000) in chromatin regions that are preferentially near or interacting with lamin-B1 based on cTSA-seq in the 90m early G1 cells was over twice the number of those (∼2000) seen in later 180m G1, and the asynchronous G1, S and G2 cells (Supplementary Figure S3C). However, we did not observe the transcription of genes, as assessed from SLAM-seq, that were transiently seen in non-LAD regions in early G1 before returning to LADs-like regions in later and asynchronous G1. This suggests that transient NL-chromatin interactions have little to no effect on gene expression in early G1.

As previously described (7,8), the vast majority of LADs mapped by cTSA-seq were stable across the G1, S and G2 stages of the cell cycle. However, minor variations in LADs across different cell cycle stages were apparent in our cTSA-seq data, as previously described in studies of cell cycle LADs using the APEX2-ID (8) and pA-DamID methods (7). These minor cell cycle-associated variable LADs (‘variable’) were H3K27me3 enriched and low in lamin-B1 signal while ‘shared’ LADs which were stable across the cell cycle were high in both H3K9me3 and lamin-B1 signal (Supplementary Figure S3D).

Thus, cTSA-seq confirms the observation of enriched distal chromosome interaction at or near the NL in early G1, and extends this to the HCT116 cells.

### The similarity of NL-chromatin relationships between early G1 cells and Oncogene-induced Senescence cells

The strong enrichment of lamin-B1 cTSA-seq signals toward chromosome ends in two different cell types in early G1 phase promoted us to look for similar patterns in all reported maps of NL-chromatin interactions. We noticed that LADs mapped by DamID in Oncogene-Induced Senescence (OIS) Tig3 human fibroblasts (30) showed a bias toward chromosome ends similar to what we observed in our lamin-B1 cTSA-seq in 90m early G1 cells (Supplementary Figures S4A, B). A second study examining RAS^V12^-induced OIS in IMR90 cells also noted a reduction of lamin-B1 chromatin immunoprecipitation (ChIP)-seq signal in the central region of chromosomes (Supplementary Figures S4C, D) (31). The signal at the distal ends of chromosomes was significantly different in early G1 (90m G1) data when compared to both later G1 (180m G1) and asynchronous G1 samples, and between the control and OIS samples from the senescence studies (Supplementary Figure S4E). Further, we observed that OIS LADs were better correlated with NL-chromatin mapped from early (90m) G1 cells than control samples (Supplementary Figure S4F). It is tempting to speculate that these OIS cells were arrested at a relatively early G1 pattern of NL-chromatin relationship.

Here, we adapted a protocol based on TSA-seq, which we call cTSA-seq and demonstrate the ability of this method to map chromatin regions found in the B compartment of the genome that are at or near the NL in different tissue culture cells. We found that the cTSA-seq method maps most LADs (>97%) identified by TSA-seq but offers increased sensitivity by also detecting nuclear periphery heterochromatic regions that fall within the B-compartment defined by Hi-C. Importantly, the cTSA-seq method is capable of mapping these chromatin regions from as few as 50 000 cells, and with further technical adaptation such as the use of carriers, should be useful for much lower cell numbers such as those found *in vivo*. As a proof of principle, we applied cTSA-seq to map the previously observed dynamic proximal NL-chromatin interactions that appear during the transient early G1 stage of the cell cycle (7). The cTSA-seq method confirms the early G1 enrichment of distal ends of chromosome regions interacting with or proximal to the NL in hTERT-RPE1 cells and expands the observation to the HCT116 cell line, suggesting that this is a common feature of early G1. We note that this enrichment of proximal NL-chromatin signal at the distal ends of chromosomes in early G1 is reminiscent of those seen in OIS.

### cTSA-seq enables the mapping of NL-proximal chromatin from fixed-frozen tissue sections

The mapping of NL-chromatin interactions has been primarily performed using *in vitro* cultured cells. A recently developed method, GO-CaRT, is able to map the *in vivo* state of both LADs and nuclear speckle-associated domains from freshly-isolated unfixed nuclei (22). However, there has been no method for mapping using fixed tissue sections. We therefore explored the feasibility of applying cTSA-seq to this type of preserved tissue resource. We used the cerebellum of 5 month-old C57BL6 adult mice, which to our knowledge does not have mapping data on NL-chromatin interactions. The cerebellum at 5 months is mature and estimated to be composed chiefly (∼80–99%) of granule cell neurons (52,53). As such, we used the cerebellum without further isolating granule cells.

**Figure 6.**
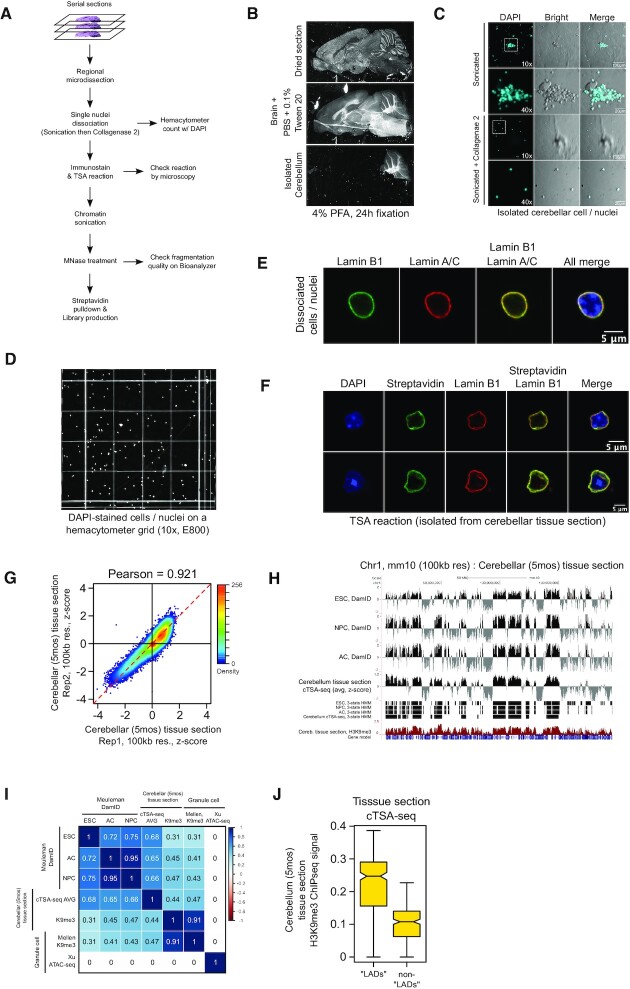
(**A**) General pipeline for the application of cTSA-seq to fixed-frozen tissue sections. Quality control steps are denoted at several points in the pipeline as horizontal arrows. (**B**) Example images of a 5 month old fixed-frozen sagittal brain section on glass slide that was thawed (top panel), incubated in PBS + 0.1% Tween-20 (middle panel) and isolated cerebellum (bottom panel). (**C**) Example images of isolated cells/nuclei after brief sonication (top two rows) and after sonication plus treatment with Collagenase Type 2 (bottom two rows). The images are of DAPI (cyan, left column), bright-field (middle column) and a merge of the two (right column). The objective used is denoted in the DAPI panel for each row. 40x objective insets are boxed in the 10× image. (**D**) Example images of the DAPI stained cells/nuclei on a hemocytometer grid used for cell/nuclei counting. DAPI and bright-field channels were simultaneously imaged and complete quadrants were stitched into a composite image. (**E**) Images of lamin-A/C (red) and lamin-B1 (green) immunostaining for a cell/nuclei isolated from fixed-frozen 5 month old cerebellum tissue sections. DAPI is presented in blue. (**F**) Close up images of a cTSA-seq reaction verification experiment on two cells/nuclei isolated from the 5 months old mouse cerebellum. Lamin-B1 (red), DAPI (blue), and merged channels are shown for both Streptavidin and lamin-B1 and for all stains performed. (**G**) Scatter plot showing the cerebellar tissue section cTSA-seq replicates. The cTSA-seq data was mapped to mm10 at 100 kb resolution and presented as a *z*-score. The Pearson correlation (0.921) is displayed at the top of the chart. (**H**) UCSC Genome browser tracks comparing DamID mapped in cells cultured *in vitro* (top three tracks) and fixed-frozen cerebellar tissue section cTSA-seq (fourth track). The data DamID data (5,36) was obtained from and lifted over to mm10 using the UCSC Genome browser. DamID was then quantitated over 100 kb genome windows. Visualization of NL-associated chromatin regions and LADs are presented as HMM calls on tracks 5–8. H3K9me3 ChIP-seq data from fixed-frozen cerebellar tissue sections is presented in maroon as the ninth track and the gene model is presented as the tenth track. All data is presented at 100 kb resolution except the H3K9me3 ChIP-seq data, which was at 20 kb resolution. cTSA-seq data is presented as an average z-score. (**I**) Heatmap matrix showing the Pearson correlation between the DamID data, the fixed-frozen cerebellar tissue section cTSA-seq, the fixed-frozen cerebellar tissue section H3K9me3 CHIP-seq data, and the published H3K9me3 ChIP-seq (37) and ATAC-seq (38) datasets from FACS-isolated granule cells. ‘ESC’ is embryonic stem cell, ‘AC’ is astrocyte, ‘NPC’ is neural progenitor cell. (**J**) Boxplot showing the quantitation of H3K9me3 ChIP-seq data obtained from fixed-frozen cerebellar tissue sections over NL-associated chromatin features (referred to as ‘LADs’) identified by HMM calling of fixed-frozen cerebellar tissue section cTSA-seq. The notches represent the confidence interval for median difference.

We first developed a pipeline (Figure 6A) to dissociate cells or nuclei from a tissue section. We collected serial, sagittal brain sections (100 μm total) starting from the cerebellum vermis. As seen in Figure 6B, we isolated the cerebellum by removing the rest of the brain. The isolated cerebellum was then dissociated with a combination of brief sonication and enzymatic digestion with Type 2 collagenase (see Materials and Methods). The suspension was filtered through a nylon mesh to remove large pieces of unbroken tissue. As seen in Figure 6C, the filtrate appeared to mainly consist of nuclei, although what appeared to be nuclei associated with cytoplasmic material were sometimes seen (Supplementary Figure S5A). We refer to these preparations hereafter as ‘cells/nuclei’. We counted cells/nuclei on a hemocytometer with the aid of DAPI staining to differentiate from debris (Figure 6D) and we routinely obtained ∼100 000 cells/nuclei per ∼100 μm of cerebellar tissue section. The isolated cells/nuclei from fixed-cerebellum tissue sections could be immunostained with both lamin-A/C and lamin-B1 antibodies (Figure 6E). The TSA reaction could be performed with isolated cell/nuclei as described for culture cells without any modifications (Figure 6F).

A major hurdle in the application of cTSA-seq to tissue sections is the inefficient fragmentation of chromatin that has been fixed for 16–24 h. To overcome this, we employed a two-step approach (Materials and methods) that uses an initial sonication of chromatin to ∼10 kilobases followed by an MNase digest, which resulted in fragmentation of chromatin to a final size range of 200–1000 bp (Supplementary Figure S5B). We note that the use of less destructive enzymes such as restriction enzymes commonly used in Hi-C protocols were ineffective. We used this chromatin fragmentation protocol for both cTSA-seq and ChIP-seq experiments below.

The lamin-B1 cTSA-seq data (mm10) from the 5-month-old cerebellum was consistent between biological replicates (Figure 6G, Pearson = 0.921). The tissue section cTSA-seq was both visually and statistically similar to the published *in vitro* DamID datasets generated for embryonic stem cells (ESC), astrocyte cells (AC) and neural progenitor cell (NPC) (Figure 6H, 6I) (5,36). A measurement of H3K9me3 ChIP-seq signal, which we mapped using the same fixed cerebellar tissue sections, over HMM-defined NL-associated chromatin (Figure 6H, HMM and Cerebellum tissue section H3K9me3 tracks) revealed the expected elevation of H3K9me3 signal (Figure 6J). Our H3K9me3 ChIP-seq was also well-correlated with a previously published H3K9me3 ChIP-seq dataset using FACS-isolated, adult granule cells, but not with the open chromatin mapped using the Assay for Transposase-Accessible Chromatin with high-throughput sequencing (ATAC-seq) (Figure 6I) (37,38). We note that NL-associated chromatin regions mapped by cTSA-seq from the cerebellar tissue section were larger than DamID LADs mapping in various cultured cells (Supplementary Figure S5C). Nevertheless, these results indicate that cTSA-seq is versatile for both *in vitro* culture cells and *in vivo* samples derived from fixed-frozen tissue sections.

While cerebellum development begins during embryogenesis, major milestones including the formation of most granule cells, synapse formation and structural foliation, occur postnatally (54,55). Cerebellum development is essentially complete at around one month (30–35 days) after birth (54,55). Previous efforts have also identified genes that are expressed at different stages of cerebellum development. Examples of such include the *Math1*/*Atoh1* and *Gabra6* genes. Math1/Atoh1 (Atonal BHLH Transcription Factor 1) is a basic Helix-Loop-Helix transcription factor critical to the formation of cerebellar granule cells (56). Math1/Atoh1 expression is seen during embryogenesis and postnatal cerebellum development but not in adult mice (57,58). Conversely, Gabra6 (Gamma-aminobutyric acid type A receptor subunit alpha6) is expressed in post-mitotic granule cells beginning around 5 days postnatal, which is supported by observations from explanted cultures of cerebellar granule cells derived from postnatal day 6 (59–61).

**Figure 7.**
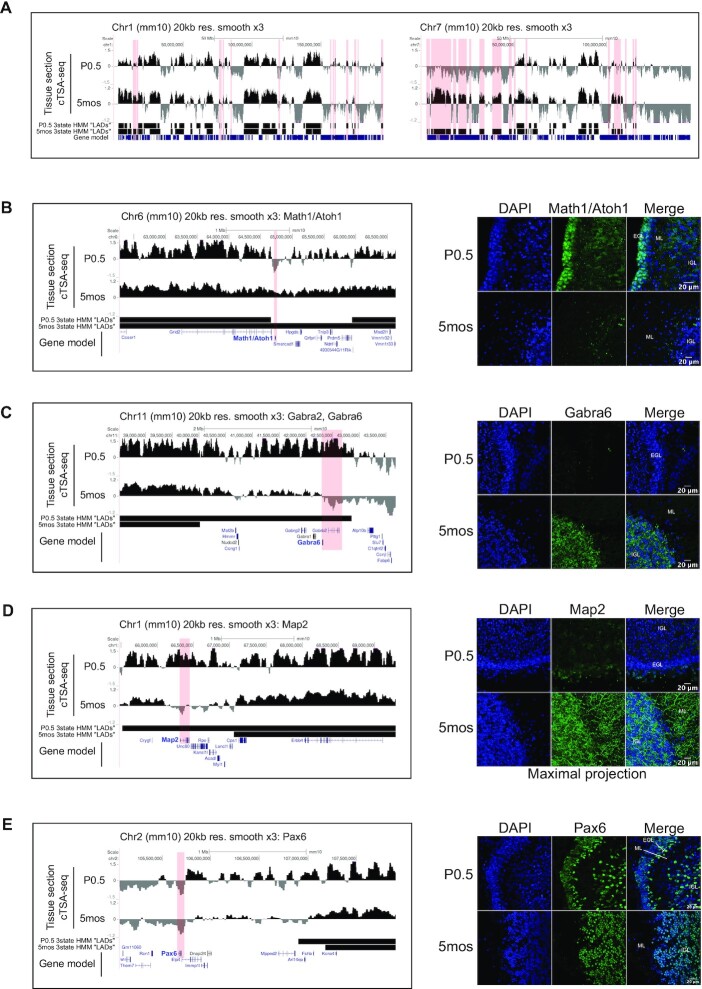
(**A**) UCSC Genome browser track (mm10) showing chromosome 1 (left panel) and chromosome 7 (right panel). Unique NL-associated chromatin regions are highlighted in light red for chromosome 7. UCSC Genome browser track showing the (**B**, left panel) Math1/Atoh1, (**C**, left panel) Gabra6, (**D**, left panel) Map2 and (**E**, left panel) Pax6 loci. The top two tracks in each panel are the average cTSA-seq data obtained from P0.5 and 5 month old cerebellum tissue sections, the third and fourth tracks represent the 3-state HMM calls (20 kb resolution) for NL-associated chromatin (‘LADs’) and the fifth track represents the gene model. The locus of interest is highlighted by a light red box and the gene name of interest is in bold and colored blue. The cTSA-seq data was mapped at 20 kb resolution and a simple moving average smoothing window of 3 was applied over the data. Left panels, representative immunostaining images of indicated brain tissue sections with a focus on the cerebellum for (B, right panel) Math1/Atoh1, (C, right panel) Gabra6, (D, right panel) Map2 and (E, right panel) Pax6. Stains for DAPI (blue), the indicated antibody (green) and a merge of the two is presented. Map2 staining is presented a maximal projection to more completely show dendritic projections. ‘EGL’ is the external granule cell layer, ‘ML’ is the molecular layer and ‘IGL’ is the internal granule cell layer.

It has been postulated that LADs may change to facilitate specific gene expression programs associated with cell differentiation and organismal development (5,22,36). This notion is indirectly supported by genome organization studies where A/B compartment switching and the coalescence of or strengthening of TAD-TAD interactions within the B-compartment were observed upon *in vitro* cell differentiation (62–64). However, direct *in vivo* evidence for LADs change is lacking, although some evidence for *in vivo* regional differences exists (22). To see if cTSA-seq could be used to identify NL-associated chromatin change during cerebellum development, we performed cTSA-seq on cells/nuclei isolated from postnatal day 0.5 (P0.5) cerebellum. The P0 cerebellum was reported to contain ∼80% cells that are either granule neuronal cells or granule neuronal cell precursors, which is similar to the percentage of the granule neuronal cells reported for adult stages (52). The P0.5 cTSA-seq NL-associated chromatin profiles were obtained from ∼100 000 cells. P0.5 cTSA-seq replicates were correlated with each other (Supplementary Figure S5D, Pearson = 0.8). The averaged P0.5 cTSA-seq data was similar to the 5 month cTSA-seq data (Figure 7A, Supplementary Figure S5E, Pearson = 0.704). Most HMM-defined NL-associated chromatin regions (at 100 kb resolution) were shared between these two time points with 89% of P0.5 regions being shared with 5 month old and 75% of 5-month-old being shared with P0.5 data. The median size of NL-associated chromatin regions from P0.5 was ∼1.5MB and smaller than those observed from 5 months (2.2MB) (Supplementary Figure S5F). The coverage of P0.5 and 5-month NL-associated chromatin regions was 33.1% and 37.9% of the genome, respectively (Supplementary Figure S5G).

We identified a number of unique NL-associated chromatin regions (Figure 7A, highlighted by red bars). P0.5 had 97 unique NL-associated chromatin regions while 64 regions were identified at 5 months. These unique regions were smaller with a median size of 0.8MB for P0.5 and 1.3MB for 5-month only regions (Supplementary Figure S5F). The genome coverage of these unique regions was between 101–112 MB (Supplementary Figure S5G). Unique NL-associated chromatin regions were found throughout the genome with some chromosomes showing a strong bias at P0.5 or 5-month (Supplementary Figure S5H). To gain further insight, we analyzed gene ontology (GO) for loci within the unique P0.5 NL-associated chromatin regions and found that the significant GO terms related to neuronal projection and synaptic formation (Supplementary Figure S5I top panel). For the 5-month unique NL-associated chromatin regions, the significant GO terms were skewed toward those involved with the immune system, with minor representation for thyroid and liver function (Supplementary Figure S5I bottom panel). These findings support the idea that global remodeling of NL-associated chromatin during cerebellum development could support gene expression changes.

To further examine if the developmental NL-chromatin association changes correlate with gene expression changes, we mapped the cTSA-seq data at 20 kb resolution and applied a moving average window of 3 to smooth the data. As seen Figure 7B (left panel), the *Math1*/*Atoh1* gene, which is known to be expressed in postnatal but not adult cerebellum (57,58), resides in a non-NL-associated chromatin region at P0.5 but became part of the NL-associated chromatin region at 5 months (compare ‘P0.5’ and ‘5mos’ tracks). This corresponded to the lack of protein expression in the adult mouse cerebellum (Figure 7B, right panel) (58). The genes for microtubule associated protein, *Map2*, and *Gabra6*, which are known to be silenced during cerebellum development and expressed during adulthood, appeared to be in NL-associated chromatin regions at P0.5, but were found outside of these regions at 5 months of age (Figure 7C and D left panels). Our immunostaining showed that the gene expression changes for *Map2* and *Gabra6* are concordant with their protein expression change at P0.5 and 5 month (Figure 7C and D, right panels). These data are consistent with previous reports on the expression of the *Gabra6* and *Map2* genes (59–61,65,66). Finally, Pax6, a transcription factor expressed in both postnatal and adult cerebellar granule cells, was seen outside of NL-associated chromatin at both P0.5 and 5 months (Figure 7E, left panel). *Pax6* gene expression corresponded to its protein expression at both time points (Figure 7E, right panel) (67).

The cTSA-seq method presented in this manuscript is an expanded iteration of the TSA-seq method that enables the mapping of nuclear peripheral chromatin from low numbers of *in vitro* and *in vivo* isolated cells. The application of cTSA-seq to fixed tissue sections reveals both global and local changes in NL-associated chromatin during cerebellum development and provides evidence for the influence of the nuclear lamina on the expression of some genes during organogenesis. The method opens the door for mapping of chromatin at the nuclear periphery from various tissues, which will aid the understanding of how the NL regulates development, aging, and disease.

## DATA AVAILABILITY

All sequencing data is available at GEO GSE186503. UCSC Genome Browser tracks are available at the following links:

Figures 1E, 2A and Supplementary Figure S1D: https://genome.ucsc.edu/s/tranjoseph/hg19_K562_Figures_1%262.

Figure 3A and Supplementary Figure S1N: https://genome.ucsc.edu/s/tranjoseph/hg19_low_cell_HCT116_cTSAseq_Figure_3.

Figure 5A, C, F, Supplementary Figures S4A, S4C: https://genome.ucsc.edu/s/tranjoseph/hg19_R90_R180_G1SG2_Lenain_Sadaie_RPE_FIgure_5%26S4.

Supplementary Figure S3B: https://genome.ucsc.edu/s/tranjoseph/hg19_R90_R180_Async_G1_rank_check_Figure_S3.

Figure 6H: https://genome.ucsc.edu/s/tranjoseph/mm10_B6_cerebellum_Reps1_2.

Figure 7: https://genome.ucsc.edu/s/tranjoseph/mm10_B6_TS_P0.5_5m_cTSAseq.

## Supplementary Material

gkac762_Supplemental_Files
