# Supplementary material for: High quality mapping of chromatin at or near the nuclear lamina from small numbers of cells reveals cell cycle and developmental changes of chromatin at the nuclear periphery
Source: Nucleic Acids Res. 2022 Sep 21;50(20):e117. doi: 10.1093/nar/gkac762 (PMC9723609; doi:10.1093/nar/gkac762)
Supplement: gkac762_Supplemental_Files [file gkac762_supplemental_files.zip › 2021_Supplemental_cTSA-seq_protocol.pdf]

cTSA-seq protocol : Zheng lab

Materials:

Chemicals:
1. Hydrogen Peroxide 35% solution (Sigma #H3410-500ML)
2. 16% paraformaldehyde (Electron Microscopy Services, #15710)
3. Glycine (Sigma, #G8898-1KG)
4. Tris-base (Roche #10708976001)
5. Sodium chloride (NaCl, EMD #SX0420-3)
6. Potassium chloride (KCl, EMD #PX1405-1)
7. EDTA (Fisher Chemical #S311)
8. IGEPAL-CA630 (Sigma #I3021)
9. Urea (Mallinckrodt #7729)
10. Sodium azide (Sigma #S2002)
11. Tween-20 (Sigma, #P5927-500ML)
12. Triton X-100 (Sigma, #T8787-250ML)
13. Sodium deoxycholic (Sigma #D6750)
14. Sodium ascorbate (Sigma #PHR1279-1G)
15. Trolox, (+-)-6hydroxy-2,5,7,8 tetramethyl chromane-2 carboxylic acid (Sigma, #23813-1G)
16. Bovine serum albumin (BSA, Sigma #A7906)
17. Goat serum (Sigma, G9023-10ML)

Prepared solutions:
1. 10x PBS and 1x PBS pH 7.4 solutions
2. 1% Hydrogen peroxide solution in 1x PBS
3. 4% Paraformaldehyde solution in 1x PBS (prepare fresh)
4. PBS + 0.01% Tween-20
5. PBS + 0.25% Triton X-100
6. 2.5M glycine solution in water
7. Blocking solution: 1x PBS + 10% BSA/normal goat serum + 10mM NaN_3 + 0.1% Triton X-100 (prepare fresh)
8. Neutralization solution: 1x PBS + 10mM sodium ascorbate + 10mM Trolox + 0.1% Tween-20 (prepare fresh)
9. RIPA buffer: 50 mM Tris, 150 mM NaCl, 0.1% (wt/vol) SDS, 0.5% (wt/vol) sodium deoxycholate and 1% (vol/vol) Triton X-100, pH 7.5) supplemented with phenylmethylsulfonyl fluoride (PMSF)
10. LiCl buffer: 250mM LiCl, 1% IGEPAL-CA630, 1% deoxycholic acid, 10mM Tris, pH 8.0, 1mM EDTA
11. High salt buffer : 1M KCl, 50mM Tris-Cl pH 8.0, 5mM EDTA
12. Urea wash buffer: 2M Urea, 10mM Tris-Cl pH 8.0

Enzymes:
1. Proteinase K: (Thermo #EO0491)

Antibody and staining reagents:
1. Rabbit anti-lamin B1 antibody (Abcam, #ab16048)
2. Streptavidin conjugate to Alexa 488 (Biolegend, #405235)
3. DAPI

Kit:
1. Biotin-XX-tyramide Superboost kit (Thermo, # B40921)

Equipment:
1. Diagenode Bioruptor Pico
2. Eppendorf Thermomixer
3. 1.5ml Diagenode Pico microtube with caps (Diagenode #C30010016)

Streptavidin pull-down and Library
1. Streptavidin beads (Pierce, #88817) or Thermo Streptavidin MyOne C1 dynabeads (Thermo #65001)
2. Ampure XP beads (Beckman #A63882)
3. DynaMag-2 (Invitrogen #12321D)
4. Takara Thruplex DNA-seq 48 kit (Takara, #R400675)

Protocol:

A. *Harvesting and fixation of cells :*
Notes: The cTSA-seq reaction can be done in solution and on a plate / coverglass. Here, the experiment is described for harvested cells in solution. Secondly, it seems essential to use non-stick tubes which should be sealed with parafilm during incubations to minimize evaporation. Centrifugation in standard Eppendorf tubes often leads to a “smearing” of cells along the wall of the tube. This smear can be collected, and is greatly reduced by using PBS with Tween-20, but I suspect that repeatedly pipetting these cells to pellet them will damage the cells to the point where the resulting sequencing data will be noisy or not work at all.

1. Wash cells with PBS and trypsinize with 2ml of 0.05% Trypsin solution.

2. Collect the trypsinized cells and add to a 15ml falcon tube containing 4ml of culture media.

3. Add in 2ml of 4% PFA and fix for 10 minutes at room temperature with gentle rocking.

4. Add in 2.5M glycine to a final concentration of 150mM (480μl) and incubate on ice for 5 minutes.

5. Pellet the cells at 300g x 5 minutes at room temperature.

6. Resuspend the cell pellet in 5ml of PBS and pellet as described in **A5**.

7. Resuspend the cell pellet in 5ml of PBS.

8. Count the fixed cells with a hemacytometer.

9. Aliquot 1 million cells and pellet at 300g x 5 minutes at room temperature. Aspirate.

B. *Permeabilization and incubation with primary antibody :*

1. Resuspend the cell pellet in PBS + 0.25% Triton X-100 and incubate at room temperature for 10 minutes.

2. Pellet at 300g x 5 minutes at room temperature.

3. Resuspend in 500μl PBS + 1% hydrogen peroxide and incubate at room temperature for 30 minutes.
    a. Leave the cap open since the pressure can cause the cap to pop off.

4. Neutralize the hydrogen peroxide with sodium ascorbate and pellet as described in **B2**.

5. Aspirate most of the solution off very carefully. Leave around 300μl. The pellet will be fluffy and is easily lost so use a P200 or P1000 for this.

6. Add in 500μl PBS + 0.1% (range of 0.001-0.1% works) Tween-20 and repeat the pelleting procedure described in **B2**.

7. Aspirate as described in **B2**.
    a. If the pellet is still not compact and at the bottom - it can be “fluffy” - repeat **B5-B7**.

8. Once aspirated completely, block the cell pellet by resuspending in blocking solution (1x PBS + 10% BSA/normal goat serum + 10mM NaN_3 + 0.1% Tween-20) and incubate for an hour at room temperature.

9. Pellet as in **B2** and aspirate.

10. Resuspend in primary antibody overnight at 4 degrees C.
    a. Let sit in a Eppendorf tube rack
    b. Antibody is diluted in blocking solution at 1:400
    c. 400μl of solution used for 1e6 cells.

C. *Secondary and TSA reaction*

1. Pellet the cells at 300g x 5 minutes at room temperature

2. Carefully aspirate off the antibody solution.

3. Wash two times with 500µl of PBS + 0.1% Tween-20 for 10 minutes each.

4. Pellet and aspirate as in **C1-C2** between each wash.

5. Wash one time with the 1x blocking solution provided with the Biotin-XX-tyramide Superboost kit. Incubate for 10 minutes and pellet as in **C1-C2**.

6. Resuspend the cells in the Anti-rabbit HRP secondary solution supplied with the Biotin-XX-tyramide Superboost kit and incubate for 2h at room temperature.
    a. Place in an Eppendorf rack in the dark.

7. Wash two times with 500µl of PBS + 0.1% Tween-20 for 10 minutes each.

8. Pellet and aspirate as in **C1-C2** between each wash.

9. Wash with 300µl 1x reaction buffer.
    a. The 1x reaction buffer is prepared from the 10x reaction buffer stock solution supplied with the Biotin-XX-tyramide Superboost kit

10. Prepare 300µl 1x reaction and add 3µl of the 100x Biotin Tyramide stock solution.
    a. The Biotin Tyramide stock solution is prepared in DMSO according to the manufacturer's suggestions.

11. Resuspend the cell pellet by adding the 1x reaction buffer + Biotin Tyramide.
    a. Do not pipet up and down if you're using standard tips and Eppendorf tubes. There is a tendency for the cells to "clump" inside the pipet tip.
    b. Instead, flick the tube gently.

12. Add in the 100x Hydrogen Peroxide solution supplied with the Biotin-XX-tyramide Superboost kit and incubate for 10 minutes at 25 degrees C in a Thermomixer.

13. Neutralize the reaction by adding 1 volume of Neutralization solution (1x PBS + 10mM sodium ascorbate + 10mM Trolox + 0.1% Tween-20).
    a. It is OK to use a non-crystalline version of sodium ascorbate (e.g., Sigma #A4034). The cell pellet, however, is slightly "brown".

14. Wash with 500µl Neutralization solution.

15. Pellet and aspirate as in **C1-C2**

16. Wash with 500µl PBS + 0.1% Tween-20. Pellet and aspirate as in **C1-C2**
    a. Check the reaction here!!
    b. Take an (~50µl) aliquot after resuspending the cells to a new 1.5ml tube
    c. Stain this by adding in 150µl PBS + 0.1% Tween-20 containing
        i. Streptavidin 488 (1:200)
        ii. Goat-anti rabbit 594 (1:600)
        iii. DAPI (1:1000)
    d. Stain for 40 minutes.
    e. Wash with PBS + 0.1% Tween-20 three times and aspirate carefully between washes.
    f. After final wash, aspirate and resuspend in 5µl of PBS + 0.1% Tween-20. Add 5µl of Prolong anti-fade, mount on a slide and image.

17. Aspirate. Store at -80 degrees C or proceed ahead to the sonication and pull-down procedure.

D. *Lysis, sonication and pull-down*

1. If the cells were frozen, thaw on ice for 15 minutes.

2. Resuspend the pellet in 600μl RIPA buffer supplemented with PMSF.

3. Lyse at 4 degrees C with rotation for 30 minutes.

4. Dispense 300μl of the lysed material into two 1.5ml Diagenode Pico tubes.

5. Sonicate
    a. Sonicate for 30 minutes total : 30 seconds on/off.
    b. We determined this empirically and chose a length of time where the DNA was sheared to around 200-400bp. You need to do this for each cell type.

6. Pellet the sonicated material at 12,000g x 30 seconds at 4 degrees C.

7. Collect 1/10th (60μl) of the lysate for input.
    a. Add 10μl Proteinase K solution
    b. Incubate at 50 degrees C with 700rpm shaking in an Eppendorf Thermomixer.

8. Collect the remaining clarified lysate to a new 1.5ml tube (~540μl).

9. Add in 410μl of RIPA buffer supplemented with PMSF.

10. Prepare the Streptavidin magnetic beads by dispensing 75μl to a microfuge tube.
    a. Magnetize on a Dyna-Mag2, aspirate and wash with 150μl of RIPA + PMSF
    b. Magnetize and aspirate.
    c. Resuspend in 50μl of RIPA + PMSF.

11. Add 50μl of the Streptavidin / RIPA + PMSF slurry and add to the clarified lysate.

12. Rotate overnight at 4 degrees C.

E. *Streptavidin pull-down washes and isolation of DNA*

1. Magnetize the Streptavidin pull-down on a DynaMag-2.
    a. Around 1 minute is required to tack the beads to the back of the microfuge tube.

2. Wash with 1ml of the following. Magnetize and aspirate in between.
    a. One time with 1ml RIPA + PMSF. Incubate on ice for 2 minutes.
    b. One time with 1ml RIPA + PMSF. Incubate on ice for 2 minutes.
    c. One time with 1ml LiCl buffer. Incubate on ice for 2 minutes.
    d. One time with 1ml High Salt buffer. Briefly
    e. One time with 1ml Urea buffer. Briefly.
    f. One time with 1ml RIPA. Brielfy

3. Aspirate and resuspend the beads in 100μl RIPA buffer.

4. Add 10μl of Proteinase K. Incubate overnight at 50 degrees C with 700rpm shaking in a Eppendorf Thermomixer.

5. Isolate the input DNA with Ampure XP beads.
    a. 1:1 final ratio (60μl : 60μl).
    b. Elution of DNA with low-volumes of Buffer EB is not particularly easy especially when an excessive amount of Ampure XP beads is used.
    c. We prepared the Ampure XP buffer (20% PEG 8000, 2.5M NaCl, 10mM NaN_3) and add 45μl of this buffer and 15μl of the original Ampure XP bead slurry.

d. Incubate for 10 minutes at room temperature.
e. Magnetize and aspirate with a P200.
f. Wash two times with 80% ethanol. Aspirate between washes with a vacuum trap aspirator.
g. Air dry the beads until the "shine" disappears and elute with 12μl of Buffer EB (from the Qiagen PCR / plasmid / gel extraction kit. The buffer is 10mM Tris-Cl pH 8.5)
h. Quantitate by Qubit.

6. For DNA isolation from the Streptavidin pull-down.
   a. First magnetize the overnight Proteinase K digestion on the Dyna-Mag2.
   b. Collect the liquid and transfer to a new 1.5ml tube (no beads, please)
   c. Purify by Ampure XP as done for the input in **E5**.
      i. Here, the volume is 110μl
      ii. I add 95μl of Ampure XP buffer and 15μl of Ampure XP bead slurry.
   d. Elute with 12μl of Buffer EB and quantitate by Qubit.

7. Check the fragmentation pattern on the Bioanalyzer prior to library production.

8. Library construction is done using the Takara (was Rubicon) Thruplex kit according the manufacturer's instructions.
   a. Use 2-5ng of DNA. Scale to whichever is lower.
