# Supplementary material for: High quality mapping of chromatin at or near the nuclear lamina from small numbers of cells reveals cell cycle and developmental changes of chromatin at the nuclear periphery
Source: Nucleic Acids Res. 2022 Sep 21;50(20):e117. doi: 10.1093/nar/gkac762 (PMC9723609; doi:10.1093/nar/gkac762)
Supplement: gkac762_Supplemental_Files [file gkac762_supplemental_files.zip › 20220727_Supplemental_Figures_and_legends.pdf]

Figure S1

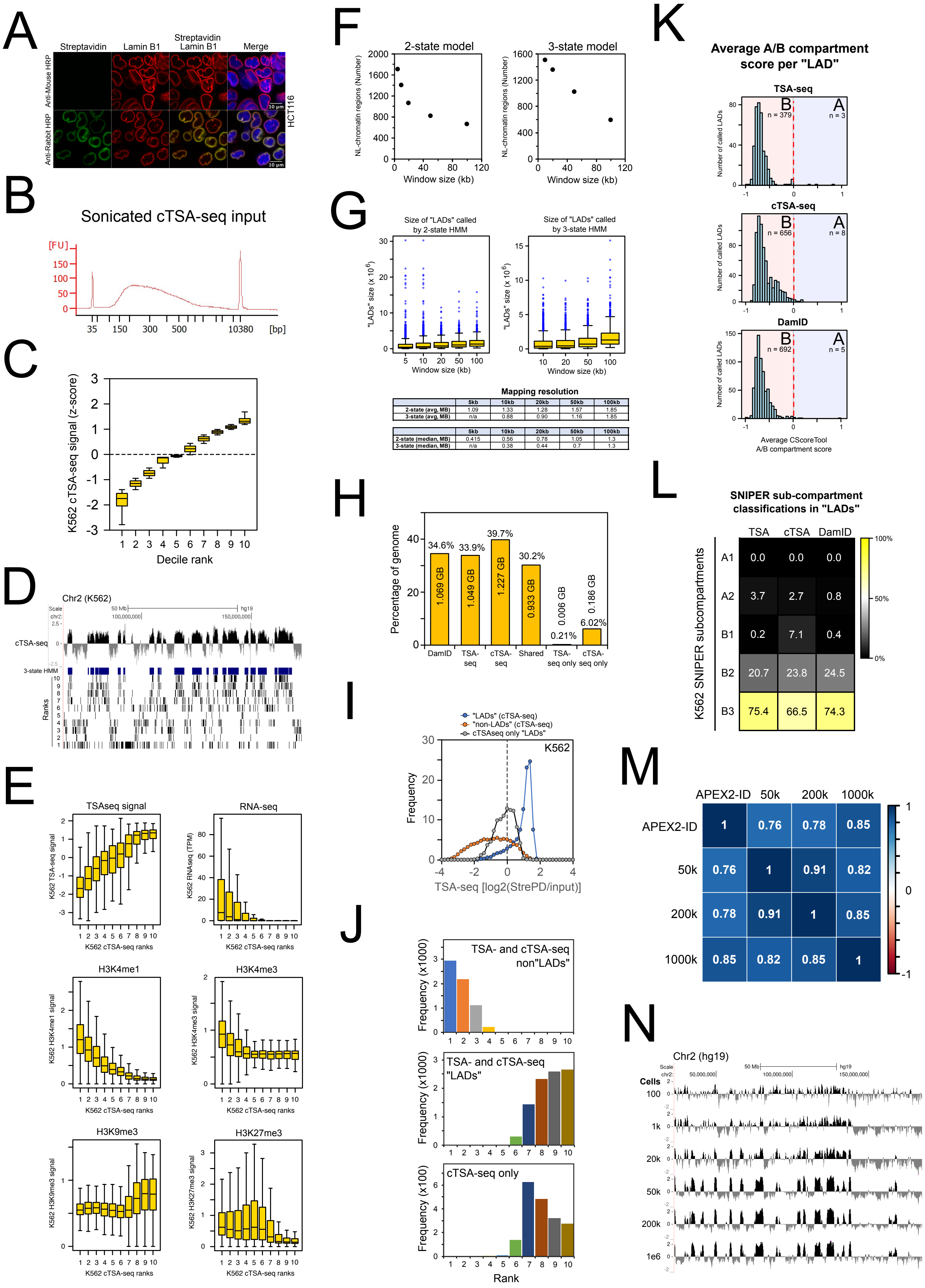

Mapping resolution

	5kb	10kb	20kb	50kb	100kb
2-state (avg, MB)	1.09	1.33	1.28	1.57	1.85
3-state (avg, MB)	n/a	0.88	0.90	1.16	1.85

	5kb	10kb	20kb	50kb	100kb
2-state (median, MB)	0.415	0.56	0.78	1.05	1.3
3-state (median, MB)	n/a	0.38	0.44	0.7	1.3

	TSA	cTSA	DamID
A1	0.0	0.0	0.0
A2	3.7	2.7	0.8
B1	0.2	7.1	0.4
B2	20.7	23.8	24.5
B3	75.4	66.5	74.3

	APEX2-ID	50k	200k	1000k
APEX2-ID	1	0.76	0.78	0.85
50k	0.76	1	0.91	0.82
200k	0.78	0.91	1	0.85
1000k	0.85	0.82	0.85	1

Figure S1: cTSA-seq labeling controls and additional details regarding cTSA-seq mapping

(A) The TSA reaction performed with HCT116 cells using an anti-rabbit HRP or an anti-mouse HRP secondary. Staining was done with Streptavidin (green) and lamin-B1 (red). **(B)** An example Bioanalyzer plot showing the distribution of DNA fragments in base pairs ("bp") obtained after sonication. **(C)** Boxplot showing the distribution of K562 cTSA-seq signal in decile ranks. **(D)** UCSC Genome browser view (hg19) showing the location of decile ranks on chromosome 2. The decile ranks are shown in descending order with the strongest cTSA-seq signal in rank 10 and the weakest in rank 1. The 3-state HMM track representing the called cTSA-seq NL-associated chromatin regions ("cTSA-seq HMM") is shown in dark blue. **(E)** Boxplots showing the quantification of K562 TSA-seq (by lamin-B 2D8 antibody) ("TSA-seq signal"), RNA transcription ("RNA-seq"), euchromatin ("H3K4me1" and "H3K4me3") and heterochromatin ("H3K9me3" and "H3K27me3") signals across cTSA-seq deciles. **(F)** Plots showing the number of cTSA-seq NL-associated chromatin regions identified using the 2-state and 3-state HMM algorithms according to window sizes (mapping resolution). **(G)** Boxplot showing the size of NL-associated chromatin regions identified by the 2-state and 3-state HMM algorithms at different resolutions. The blue dots represent the outliers. The bottom table shows the average and median length of NL-chromatin regions identified by the HMM calls. **(H)** Bar chart showing the genomic coverage of NL-associated chromatin mapped by DamID, lamin-B TSA-seq and cTSA-seq as well as the coverage for regions shared between TSA- and cTSA-seq, TSA-seq only and cTSA-seq only. The gigabases (GB) and percentage of the genome covered are shown on the graph. **(I)** Line plot showing the TSA-seq signal associated with different cTSA-seq features. We examined this signal over cTSA-seq defined NL-associated chromatin "LADs" (blue), "non-LADs" (orange) and "cTSA-seq only LADs" (grey). **(J)** Bar charts showing the cTSA-seq decile ranking of regions classified as shared nonLADs (top), shared "LADs" (middle) and cTSA-seq only "LADs" (bottom). **(K)** Histogram showing the average K562 A/B compartment score over TSA-seq (using lamin-B 2D8 antibody, top panel), cTSA-seq (middle panel) and DamID (bottom panel). The A/B compartment score was calculated from K562 Hi-C data using the CscoreTool program and these scores were averaged over the identified NL-chromatin region coordinates. The x-axis represents the average A/B compartment score and the y-axis represents the number of LADs. The red-dashed line is the zero line. **(L)** A heatmap of SNIPER K562 Hi-C A/B sub-compartment composition for TSA-seq, cTSA-seq and DamID mapped LADs. The SNIPER A/B sub-compartment classifications at 100 kb resolution were obtained from Xiong and Ma., 2019 and then intersected with LADs coordinates. The composition was calculated as a percentage of the total number of intersected sub-compartment classifications. **(M)** A heatmap showing the Pearson correlation between cTSA-seq (using the indicated number of cells) and APEX2-ID in HCT116 cells. (**N**) UCSC genome browser view (hg19) of HCT116 cTSA-seq signal (z-score) for indicated numbers of cells. Each data set is an average of two biological repeat, except for the 100-cell experiment which is from a single experiment.

Figure S2

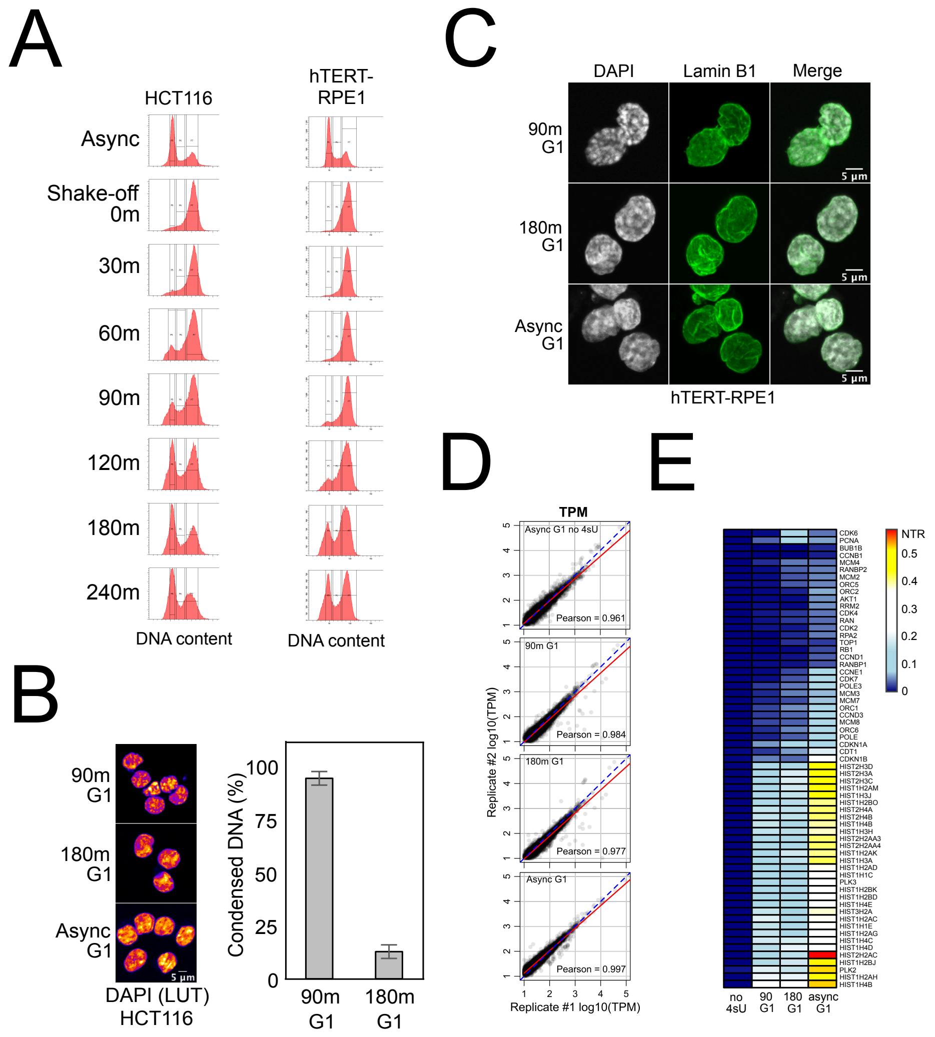
A
HCT116
hTERT-RPE1
Async
Shake-off 0m
30m
60m
90m
120m
180m
240m
DNA content
DNA content
B
90m G1
180m G1
Async G1
DAPI (LUT) HCT116
5 µm
Condensed DNA (%)
100
75
50
25
0
90m G1
180m G1
C
DAPI
Lamin B1
Merge
90m G1
180m G1
Async G1
5 µm
hTERT-RPE1
D
TPM
Async G1 no 4sU
Pearson = 0.961
90m G1
Pearson = 0.984
180m G1
Pearson = 0.977
Async G1
Pearson = 0.997
Replicate #2 log10(TPM)
Replicate #1 log10(TPM)
E
CDK6
PCNA
BUB1B
CCNB1
MCM4
RANBP2
MCM2
ORC5
ORC2
AKT1
RRM2
CDK4
RAN
CDK2
RPA2
TOP1
RB1
CCND1
RANBP1
CCNE1
CDK7
POLE3
MCM3
MCM7
ORC1
CCND3
MCM8
ORC6
POLE
CDKN1A
CDT1
CDKN1B
HIST2H3D
HIST2H3A
HIST2H3C
HIST1H2AM
HIST1H3J
HIST1H2BO
HIST2H4A
HIST2H4B
HIST1H4B
HIST1H3H
HIST2H2AA3
HIST2H2AA4
HIST1H2AK
HIST1H3A
HIST1H2AD
HIST1H1C
PLK3
HIST1H2BK
HIST1H2BD
HIST1H4E
HIST3H2A
HIST1H2AC
HIST1H1E
HIST1H2AG
HIST1H4C
HIST1H4D
HIST2H2AC
HIST1H2BJ
PLK2
HIST1H2AH
HIST1H4B
NTR
0.5
0.4
0.3
0.2
0.1
0
no 4sU
90 G1
180 G1
async G1

Figure S2: Quality control and features from early G1 FACS and SLAM-seq experiments

(A) Typical FACS profiles showing the time course of emergence of G1 cells after nocodazole release. DNA was stained with Hoechst 33342 prior to FACS. Asynchronous populations ("Async") were used as a gating reference. The left and right panels are representative experiments done for HCT116 and hTERT-RPE1 cells, respectively. **(B)** Left panel shows a look up table (LUT) display of DAPI signal (DNA) for FACS sorted early G1 (90m G1), later G1 (180m G1) and asynchronous G1 (Async G1) HCT116 cells. Right panel shows a graph of the percentage of cells containing apparently dissimilar chromatin in the 90m early G1 population when compared to 180m G1 populations. **(C)** Lamin-B1 immunostaining for FACS isolated early ("90m G1"), later ("180m G1") and asynchronous G1 ("Async G1") hTERT-RPE1 cells. Lamin-B1 is presented in green and DNA (DAPI) is presented as grayscale. The scale bar represents 5 microns. **(D)** Scatter plots for SLAM-seq replicates. The axes are a log10 transformation of the transcripts per million (TPM). Untreated asynchronous G1 ("Async G1 no 4sU"), early ("90m G1"), later ("180m G1") and asynchronous G1 ("Async G1") HCT116 populations were examined. The blue dashed line is a reference diagonal line and the red line represents a linear model. The Pearson correlation value is presented in each panel on the lower right. **(E)** Heatmap showing the new transcription (NTR) level for select genes, including known cell cycle proteins and histone gene clusters, in the indicated cell populations. Note that the asynchronous G1 with no 4sU is labeled as "no 4sU".

Figure S3

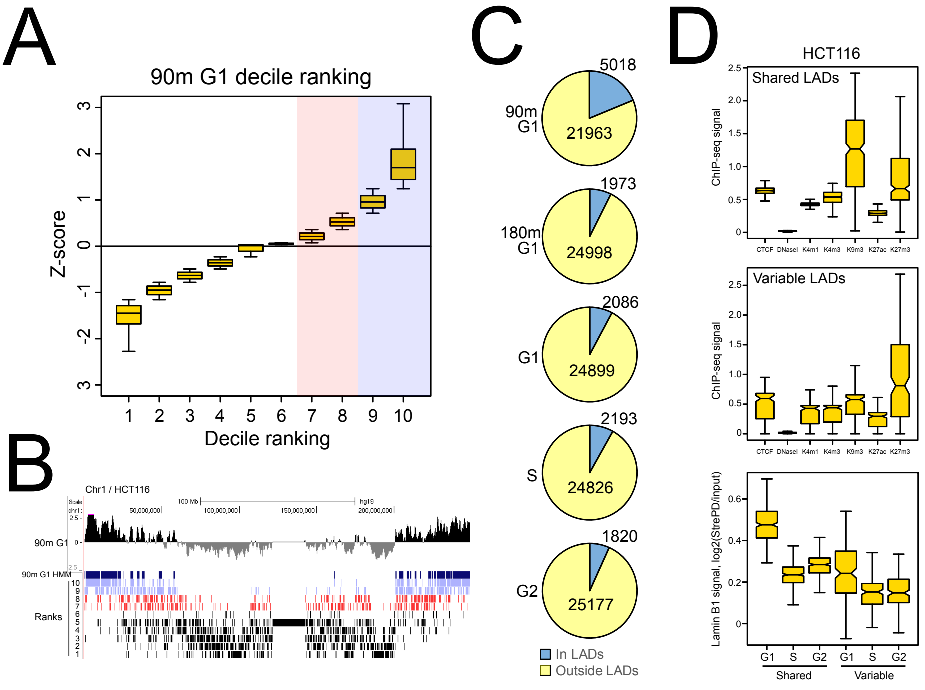

A
90m G1 decile ranking
Z-score
Decile ranking
B
Chr1 / HCT116
90m G1
90m G1 HMM
Ranks
C
90m G1
5018
21963
180m G1
1973
24998
G1
2086
24899
S
2193
24826
G2
1820
25177
In LADs
Outside LADs
D
HCT116
Shared LADs
Variable LADs
ChIP-seq signal
CTCF
DNaseI
K4m1
K4m3
K9m3
K27ac
K27m3
Lamin B1 signal, log2(StrePD/input)
G1
S
G2
Shared
Variable

Figure S3: Decile ranking and additional analyses of cell cycle-related cTSA-seq experiments

(A) Example boxplot showing the decile ranking of cTSA-seq z-score data for the 90m G1 sample. The light red bar highlights a weak positive cTSA-seq z-score while the blue bar represents the strongest cTSA-seq z-score. **(B)** Example UCSC Genome Browser view (Chr1, hg19) showing the location of decile ranks for 90m G1 cells and their relationship to the HMM calls. The HMM call for NL-chromatin regions at 90m G1 is shown in dark blue. The 9th and 10th ranks, which correspond to the strongest cTSA-seq signal are shown in light blue and the 7th and 8th ranks are shown in red.
(C) Pie-charts showing the number of genes inside and outside of the lamin-B1 cTSA-seq mapped regions (referred to here as “In LAD” and “Outside LAD”, respectively). Gene localization was called if at least 80% of the gene resided within the examined feature. **(D)** Boxplots showing the ENCODE HCT116 epigenetic ChIP-seq signal quantified over lamin-B1 cTSA-seq mapped chromatin regions that were shared (top panel, referred to as “Shared LADs”) or variable (middle panel, referred to as “Variable LADs”) across the G1, S, and G2 phases of the cell cycle. A boxplot showing the change in lamin-B1 cTSA-seq signal in shared and variable LADs over the cell cycle (bottom panel).

Figure S4

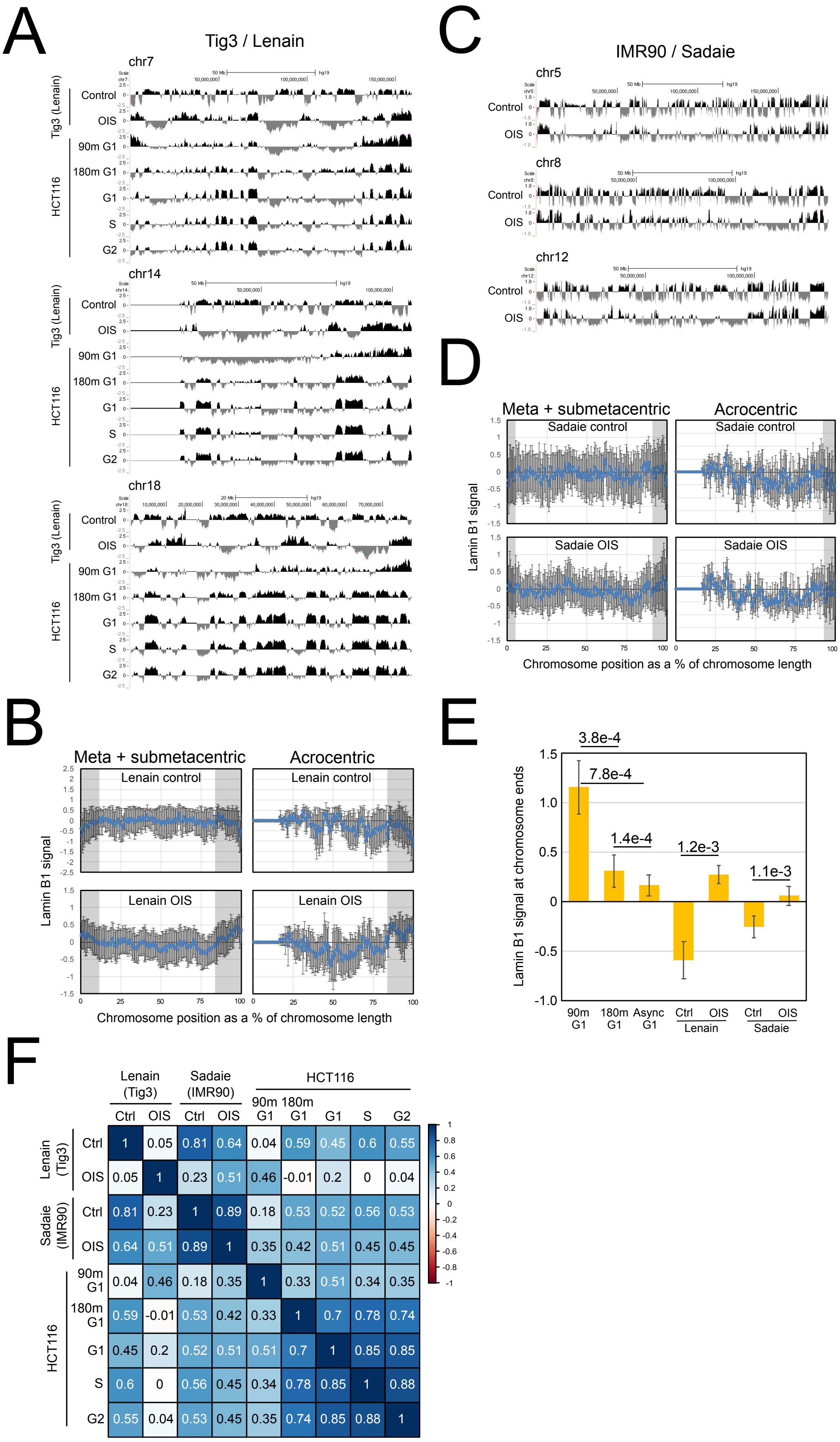
A
Tig3 / Lenain
chr7
chr14
chr18
Tig3 (Lenain)
HCT116
Control
OIS
90m G1
180m G1
G1
S
G2
B
Meta + submetacentric
Acrocentric
Lenain control
Lenain OIS
Lamin B1 signal
Chromosome position as a % of chromosome length
C
IMR90 / Sadaie
chr5
chr8
chr12
Control
OIS
D
Meta + submetacentric
Acrocentric
Sadaie control
Sadaie OIS
Lamin B1 signal
Chromosome position as a % of chromosome length
E
Lamin B1 signal at chromosome ends
3.8e-4
7.8e-4
1.4e-4
1.2e-3
1.1e-3
90m G1
180m G1
Async G1
Ctrl
OIS
Lenain
Ctrl
OIS
Sadaie
F
Lenain (Tig3)
Sadaie (IMR90)
HCT116
Ctrl
OIS
Ctrl
OIS
90m G1
180m G1
G1
S
G2
1 0.05 0.81 0.64 0.04 0.59 0.45 0.6 0.55
0.05 1 0.23 0.51 0.46 -0.01 0.2 0 0.04
0.81 0.23 1 0.89 0.18 0.53 0.52 0.56 0.53
0.64 0.51 0.89 1 0.35 0.42 0.51 0.45 0.45
0.04 0.46 0.18 0.35 1 0.33 0.51 0.34 0.35
0.59 -0.01 0.53 0.42 0.33 1 0.7 0.78 0.74
0.45 0.2 0.52 0.51 0.51 0.7 1 0.85 0.85
0.6 0 0.56 0.45 0.34 0.78 0.85 1 0.88
0.55 0.04 0.53 0.45 0.35 0.74 0.85 0.88 1

Figure S4: The early G1 chromatin at or near the reassembling NL identified by lamin-B1 cTSA-seq may be a feature present in Oncogene-Induced Senescence (OIS) cells

(**A**) UCSC Genome browser tracks (hg19) of LADs mapped by lamin-B1 DamID from "Control" and Oncogene-induced senescence ("OIS") human Tig3 cells (30) and by lamin-B1 cTSA-seq from "90m G1", "180m G1", "G1", "S" and "G2" HCT116 cells. Metacentric (chr 7), acrocentric (chr 14) and small metacentric (chr 18) chromosomes are shown. The cTSA-seq data presented are average z-scores. (**B**) Line plots displaying the average lamin-B1 DamID signal from control and OIS Tig3 cells. Meta- and submetacentric (left) and acrocentric (right) chromosomes are shown. The error bars represent standard deviation. Grey bars highlight regions where the signal is elevated in OIS samples. (**C**) UCSC Genome browser tracks for lamin-B1 ChIP-seq signal (hg19) from "Control" and "OIS" IMR90 human cells (31). Chromosomes 5, 8 and 12 are presented. (**D**) Line plots displaying the average lamin-B1 ChIP-seq signal from control and OIS IMR90 cells (31). Grey bars highlight regions where the signal is elevated in OIS cells. (**E**) Average HCT116 lamin-B1 cTSA-seq signal measured from the ends of chromosomes for 90m G1, 180m G1, asynchronous G1. The control ("Ctrl") and OIS data are from previously published work shown in A-D (30,31). We defined the chromosome ends as the last 2.5% of each chromosome arm. The p-values represent are a two-tailed t-test and error bars represent the standard deviation of the signal. (**F**) A heatmap showing the Pearson correlation between control and oncogene-induced senescence (OIS) LADs maps from Lenain, et al. (Tig3), and Sadaie et al. (IMR90), and LADs mapped for early G1 (90m G1), later G1 (180m G1), asynchronous G1, S and G2 HCT116 cells.

Figure S5

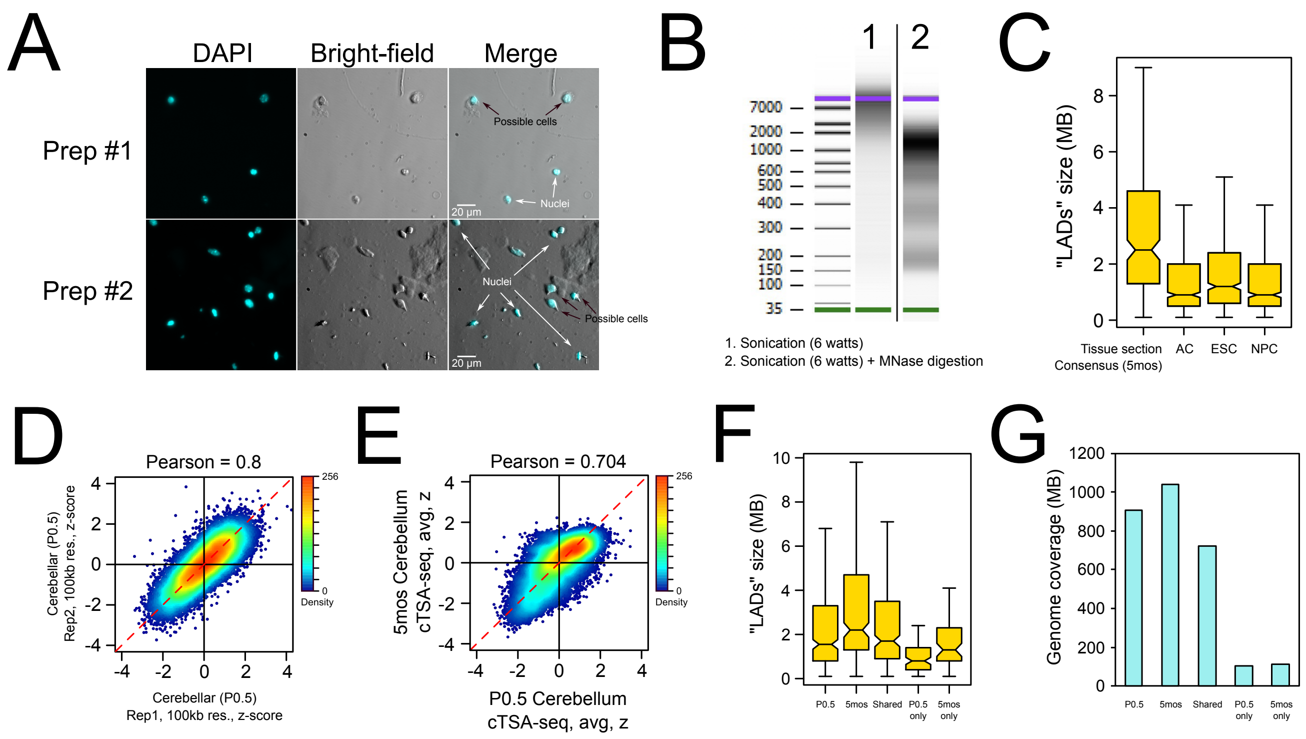

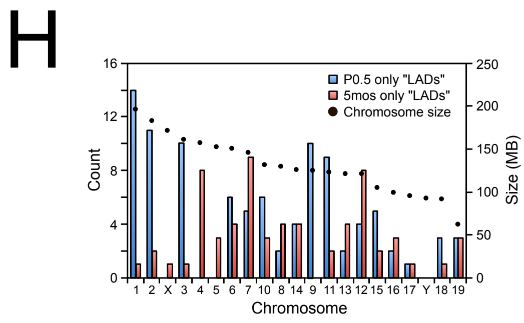

I

Genes found in P0.5 only NL-associated chromatin regions

GO biological process complete	Fold Enrichment	raw P value	FDR
plasma membrane phospholipid scrambling	18.58	1.70E-05	3.36E-02
cartilage development	4.73	3.72E-05	3.91E-02
regulation of synapse organization	3.97	2.13E-05	3.73E-02
regulation of neuron projection development	2.99	8.18E-06	2.58E-02
behavior	2.58	2.29E-05	3.60E-02
regulation of anatomical structure morphogenesis	2.25	5.69E-05	4.72E-02

Genes found in 5 month only NL-associated chromatin regions

GO biological process complete	Fold Enrichment	raw P value	FDR
plasmacytoid dendritic cell antigen processing and presentation	23.63	2.32E-05	1.45E-03
negative regulation of transposon integration	23.63	6.91E-08	6.81E-06
stimulatory C-type lectin receptor signaling pathway	17.72	5.46E-08	5.56E-06
antifungal innate immune response	13.9	5.22E-08	5.34E-06
ethanol catabolic process	12.89	3.71E-05	2.18E-03
epoxygenase P450 pathway	12.47	2.15E-13	5.21E-11
detection of chemical stimulus involved in sensory perception of bitter taste	11.81	3.75E-16	1.44E-13
3'-phosphoadenosine 5'-phosphosulfate metabolic process	11.81	5.38E-05	2.98E-03
innate immune response in mucosa	10.93	1.06E-19	7.92E-17
sodium-independent organic anion transport	9.19	4.50E-05	2.57E-03
antigen processing and presentation of exogenous peptide antigen via MHC class I	9.09	6.01E-04	2.30E-02
antimicrobial humoral immune response mediated by antimicrobial peptide	6.99	6.10E-18	3.10E-15
thyroid hormone metabolic process	6.52	9.70E-05	4.93E-03
mast cell degranulation	6.08	5.68E-05	3.11E-03
bile acid and bile salt transport	5.34	7.46E-04	2.72E-02
collagen catabolic process	5.14	7.57E-05	3.99E-03
positive regulation of membrane permeability	4.97	1.95E-05	1.24E-03
cellular response to lipopolysaccharide	3.97	4.17E-11	6.79E-09
chemokine-mediated signaling pathway	3.67	1.49E-03	4.73E-02
sensory perception of smell	1.93	9.03E-09	1.04E-06
G protein-coupled receptor signaling pathway	1.74	1.56E-09	2.00E-07

Figure S5: Identifying NL-associated chromatin by lamin-B1 cTSA-seq from fixed-frozen sections of mouse cerebellum

(**A**) Representative images of cells/nuclei isolated from the cerebellum. Two independent preparations are shown (“Prep #1”, “Prep #2”). DAPI is presented in cyan and the bright-field channel in grayscale. White arrows point to nuclei and black arrows point to nuclei with possible cytoplasmic material (“possible cells”). (**B**) Bioanalyzer rendering of a DNA separating gel showing the size distribution of DNA after sonication (denoted “1”) and after sonication followed by MNase treatment (denoted “2”). (**C**) Boxplot showing the sizes (in megabases, “MB”) of NL-associated chromatin regions identified from fixed-frozen cerebellar tissue section cTSA-seq using 5 month old adult mice and DamID mapping of LADs experiment performed in the indicated cells cultured in vitro (5,36). The notches represent the confidence interval of median difference. The mapping was done at 100 kb resolution. (**D**) Scatter plot showing the relationship between postnatal 0.5 (P0.5) day cerebellum cTSA-seq replicates. The Pearson correlation (0.8) is presented at the top of the chart. (**E**) Scatter plot showing the relationship between P0.5 and 5 month old cerebellum cTSA-seq experiments. The Pearson correlation (0.704) is presented at the top of the chart. (**F**) Boxplot showing the size of the NL-associated chromatin regions mapped by cTSA-seq from P0.5 and 5 month cerebellum tissue sections at 100 kb resolution. NL-associated chromatin regions that were shared between P0.5 and 5 month, P0.5 only and 5 month only are also presented. (**G**) Bar chart showing the genomic coverage of NL-associated chromatin regions mapped by cTSA-seq from P0.5 and 5 month cerebellum tissue sections. Regions that were shared between P0.5 and 5 month, P0.5 only and 5 month only are also presented. (**H**) Bar chart showing the number (primary y-axis, “Count”) of P0.5 only (blue bars) and 5 month only (red bars) NL-associated chromatin regions across individual chromosomes. The chromosome order is based on size and in descending order. The chromosome size is represented by a black circle and belongs to the secondary y-axis (“Size, MB”). (**I**) GO term analysis for genes found in P0.5 only (top panel) and 5 month old only (bottom panel) cerebellar NL-associated chromatin regions.
